# Unusual quadruple bonds featuring collective interaction-type σ bonds between first octal-row atoms in the alkaline-earth compounds Ae
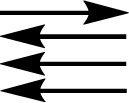
OLi_2_ (Ae = Be–Ba)†‡

**DOI:** 10.1039/d4sc01979b

**Published:** 2024-07-31

**Authors:** Li-Juan Cui, Yu-Qian Liu, Sudip Pan, Zhong-Hua Cui, Gernot Frenking

**Affiliations:** a Institute of Atomic and Molecular Physics, Jilin University Changchun 130023 China zcui@jlu.edu.cn sudip@jlu.edu.cn; b Key Laboratory of Physics and Technology for Advanced Batteries (Ministry of Education), Jilin University Changchun 130023 China; c Philipps-Universität Marburg Hans-Meerwein-Strasse 4 D-35043 Marburg Germany; d Institute of Advanced Synthesis, School of Chemistry and Molecular Engineering, Nanjing Tech University Nanjing 211816 China frenking@chemie.uni-marburg.de

## Abstract

Quantum chemical calculations are reported for the complexes of alkaline earth metals AeOLi_2_ (Ae = Be–Ba) at the BP86-D3(BJ)/def2-QZVPP and CCSD(T)/def2-QZVPPQZVPP levels. The nature of the Ae–OLi_2_ bond has been analyzed with a variety of methods. The AeOLi_2_ molecules exhibit an unprecedented σ donor bond Ae→OLi_2_ where the (*n*)s^2^ lone-pair electrons of the Ae atom are donated to vacant O–Li_2_ antibonding orbitals having the largest coefficient at lithium. This is a covalent bond where the accumulation of the associated electronic charge is located at two positions above and below the Ae–OLi_2_ axis. The bifurcated component of orbital interactions is structurally related to the recently proposed collective bonding model, but exhibits a completely different type of bonding. The most stable isomer of AeOLi_2_ has a *C*_2v_ geometry and a singlet (^1^A_1_) electronic ground state. The bond dissociation energy (BDE) of the Ae–OLi_2_ bonds exhibits a zig-zag trend from BeOLi_2_ to BaOLi_2_, with BeOLi_2_ having the largest BDE (*D*_e_ = 73.0 kcal mol^−1^) and MgOLi_2_ possessing the lowest BDE (*D*_e_ = 42.3 kcal mol^−1^) at the CCSD(T) level. The calculation of the atomic partial charges by the Hirshfeld and Voronoi methods suggests that Be and Mg carry small negative charges in the lighter molecules whereas the heavier atoms Ca–Ba have small positive charges. In contrast, the NBO and QTAIM methods give positive charges for all Ae atoms that are larger for Ca–Ba than that calculated by the Hirshfeld and Voronoi approaches. The molecules AeOLi_2_ have large dipole moments where the negative end is at the Ae atom with the polarity Ae→OLi_2_. The largest dipole moments are predicted for the lighter species BeOLi_2_ and MgOLi_2_ and the smallest value is calculated for BaOLi_2_. The calculation of the vibrational spectra shows a significant red-shift toward lower wave numbers for the Ae–OLi_2_ stretching mode in comparison to diatomic AeO. Besides the Ae→OLi_2_ σ-donor bonds there are also three dative bonds due to Ae←OLi_2_ backdonation which consist of one σ bond and two π bonds. The appearance of strong Ae→OLi_2_ σ donation leads to quadruple bonds Ae
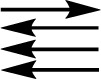
OLi_2_ in all systems AeOLi_2_, even for the lightest species with Ae = Be, Mg. The valence orbitals of Ca, Sr, and Ba, which are involved in the dative interactions, are the (*n*)s and (*n*−1)d AOs whereas Be and Mg use their (*n*)s and (*n*)p AOs. The EDA-NOCV results are supported by the AdNDP calculations which give four 2c–2e bonding orbitals. Three bonding orbitals have occupation numbers ∼2. One σ orbital has smaller occupation numbers between 1.32 and 1.73 due to the delocalization to the lithium atoms. The analysis of the electronic structure with the ELF method suggests multicenter bonds with mainly trisynaptic and tetrasynaptic basins, which also support the results of the EDA-NOCV calculations.

## Abstract 2

The bonding analysis suggest that the title compounds have three dative bonds due to triple AeOLi_2_ backdonation and one unusual Ae→OLi_2_ σ donation which leads to quadruple bonds AeOLi_2_ for all systems even for the lightest species with Ae = Be, Mg.

## Graphical abstract

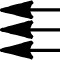

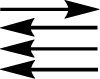

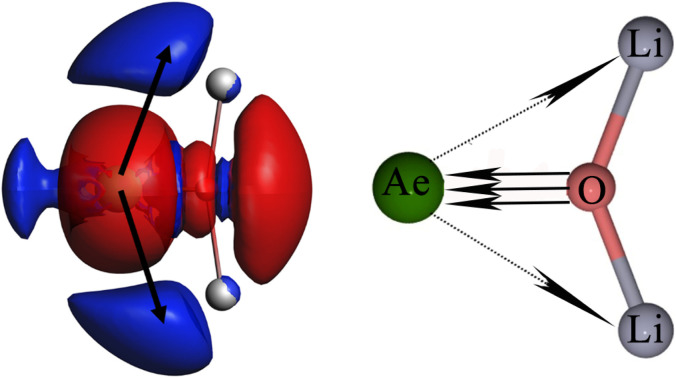

## 1. Introduction

Multiple bonds between atoms constitute a fundamental cornerstone of chemistry.^1^ While double and triple bonds are commonly encountered in molecules, compounds featuring bond orders greater than three were until recently only known for chemical bonds between transition metals and possibly actinides, because they use their valence d and f orbitals for covalent bonding.^2–5^ A previous suggestion that C_2_ possesses a quadruple bond started a vigorous controversy in the literature with many arguments both in favor^6–9^ and against.^10–15^ The controversy was finally solved by an experimental study using high-resolution photoelectron imaging spectrometry, which showed that dicarbon has a strong degenerate π bond but negligible σ bonding due to near cancellation of bonding and antibonding σ-orbital interactions like in Be_2_.^16^

Quadruple bonding of main-group atoms between boron and various transition metals (TMs) was lately reported in joint experimental and theoretical studies of molecules where the bond multiplicity of TM–B bonds can vary from single to quadruple bonding.^17^ In particular, diatomic RhB^−^ was experimentally detected and the transition to neutral RhB was studied by photoelectron spectroscopy.^18^ The analysis of the spectra and quantum chemical calculations suggests that the neutral molecule in its ^1^Σ^+^ electronic ground state possesses a quadruple bond Rh≣B which consists of two σ and two π bonds. Dative quadruple bonds between beryllium and various d^10^ transition metals were recently reported by Parameswaran.^19^

The finding that only one atom of a chemical bond A–B needs to provide valence d orbitals in order to achieve a quadruple bond led us to investigate the chemical bonds of heavy alkaline earth (Ae) atoms, Ca, Sr, and Ba with first octal-row species, because previous studies showed that these Ae atoms utilize their (*n*−1)d orbitals for covalent bonding like transition metals.^20,21^ We found that the anions AeB^−^ and AeF^−^, and neutral AeC, where Ae = Ca, Sr, Ba, have indeed two σ and two π orbitals.^22–24^ The degenerate π orbitals in AeB^−^ and isoelectronic AeC, which have only six valence electrons and a triplet (^3^Σ^−^) ground state, have singly occupied π orbitals.^22^ In contrast, the anions AeF^−^ of the heavy Ae atoms, which have ten valence electrons, possess two doubly occupied σ and two π bonding orbitals, and thus they have a genuine quadruple bond with four strong dative components Ae
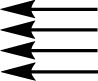
F^−^ whereas the lighter species with Ae = Be, Mg have triple bonds Ae
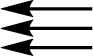
F^−^.^23,24^

In order to find neutral molecules Ae–X, which have genuine quadruple bonds, we calculated the molecules AeOLi_2_, which are valence isoelectronic with the anions AeF^−^. The calculated results yielded an unexpected finding, which is presented here. We report about quantum chemical calculations of the geometries, vibrational frequencies, and bond dissociation energies of the title compounds, and we present and discuss the results of a thorough analysis of the bonding situation using a variety of methods. The ligand species OLi_2_ was experimentally reported before,^25^ but none of the calculated adducts AeOLi_2_ is experimentally known so far. The computed Ae–OLi_2_ bond strength suggests that they can be synthesized at least in the gas phase or in low-temperature matrices. The theoretically predicted vibrational spectra are helpful to identify the molecules AeOLi_2_ experimentally.

## 2. Computational details

The exploration of potential energy surfaces (PESs) of the AeOLi_2_ (Ae = alkaline-earth atoms) systems was undertaken utilizing the CALYPSO (Crystal structure AnaLYsis by Particle Swarm Optimization) code.^26^ Initial structures for both singlet and triplet spin states were considered at the BP86-D3(BJ)/def2-SVP level,^27–30^ and subsequently refined to enhance the accuracy of geometrical and frequency predictions at the BP86-D3(BJ)/def2-QZVPP level.^30^ To attain more precise geometries and relative energies, optimizations followed by the frequency calculations at the CCSD(T)/def2-QZVPP^31,32^ level, where all electrons are correlated (full core), were conducted on the low-lying minimum energy geometries determined at the BP86-D3(BJ)/def2-QZVPP level. The reliability of the mono-determinantal methodologies employed in this study was affirmed through the attainment of small *T*_1_ diagnostic (within 0.02) values from the converged CCSD wavefunction. All of these computational investigations were executed employing the Gaussian 16 package.^33^

Chemical bonding analyses were conducted using the quantum theory of atoms in molecules (QTAIM),^34^ the adaptive natural density partitioning (AdNDP) analysis^35^ and the electron localization function (ELF),^36^ which are implemented in the Multiwfn code.^37^ The natural partial charges were evaluated using various methods, *viz.*, the natural bond orbital (NBO),^38^ QTAIM,^34^ Hirshfeld,^39^ and Voronoi^40^ approaches. NBO charge was computed using the NBO7 program,^41,42^ while Hirshfeld charges were calculated using the Gaussian 16 program. For Voronoi charges, the ADF 2020 software was used.^43^ We also calculated the bond orders using the Wiberg method^44^ as well as the Mayer approach.^45^

To gain deeper insights into the nature of chemical interactions, energy decomposition analysis (EDA)^46^ in conjunction with the natural orbital for chemical valence theory (NOCV)^47^ which leads to the combined EDA-NOCV method^48^ was carried out. This comprehensive analysis was performed at the unrestricted (U)BP86-D3(BJ)/TZ2P level^49^ where scalar-relativistic effects are considered with the ZORA method^50–52^ utilizing the ADF 2020 package.^53,54^ In this analysis, the intrinsic interaction energy (Δ*E*_int_) between two fragments is dissected into four distinct energy components, as follows:

1 Δ*E*_int_ = Δ*E*_elstat_ + Δ*E*_Pauli_ + Δ*E*_orb_ + Δ*E*_disp_

The electrostatic Δ*E*_elstat_ term represents the quasiclassical electrostatic interaction between the unperturbed charge distributions of the prepared fragments. The Pauli repulsion, Δ*E*_Pauli_ accounts for the energy change during the transformation from the superposition of unperturbed electron densities of the individual fragments into a wavefunction that explicitly adheres to the Pauli principle, achieved through the necessary antisymmetrization and wavefunction renormalization. The orbital term Δ*E*_orb_ results from the mixing of the orbitals, which causes a charge transfer between the isolated fragments and a polarization within the fragments. The dispersion contribution (Δ*E*_disp_), facilitated by the D3(BJ) method, elucidates the dispersion forces influencing the overall interaction between the fragments.

The EDA-NOCV enables the partition of the total Δ*E*_orb_ into pairwise contributions of the orbital interactions that is very important to get a complete picture of the bonding. The charge deformation Δ*ρ*_*k*_(*r*), resulting from the mixing of the orbital pairs *ψ*_*k*_(*r*) and *ψ*_−*k*_(*r*) of the interacting fragments presents the amount and the shape of the charge flow due to the orbital interactions (eqn (2)), and the associated energy term Δ*E*_orb_ provides the amount of stabilizing orbital energy originating from such an interaction (eqn (3)). Further details about the partitioning are given in the original work.^48^2
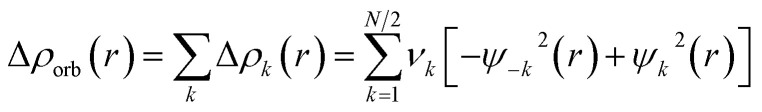
3
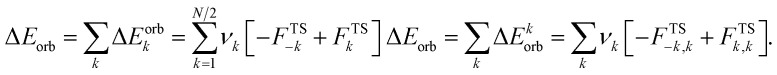


Several reviews extensively discussed details of the EDA-NOCV method and its application, offering diverse perspectives and viewpoints.^55–58^

## 3. Results

**Fig. 1:**
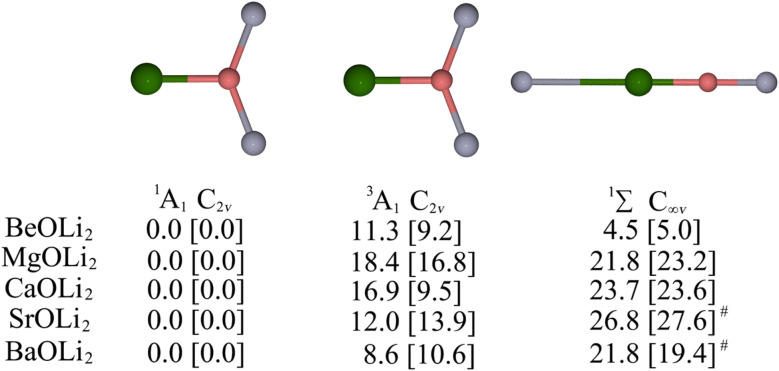
The relative energies in kcal mol^−1^ of low-lying minimum energy isomers of AeOLi_2_ (Ae = Be, Mg, Ca, Sr, Ba) computed at the CCSD(T) and BP86-D3(BJ) (in square brackets) levels with the def2-QZVPP basis set. The green, pink, and gray colors represent the Ae, O, and Li atoms, respectively. ^#^bent structure with *C*_s_ symmetry.

Fig. 1 shows the low-lying isomers of the AeOLi_2_ species which were found on the singlet and triplet potential energy surface (PES) at the BP86-D3(BJ)/def2-QZVPP and CCSD(T)/def2-QZVPP levels. Both methods predict that the *C*_2v_ structure Ae–OLi_2_ in the singlet ^1^A_1_ state is the global minimum on the PES. The linear singlet isomer (^1^Σ) with the connectivity Li–Ae–O–Li is energetically higher-lying than the *C*_2v_ form, but the energy difference for the beryllium species is not very large (4.5 and 5.0 kcal mol^−1^ at the two levels of theory). The only triplet (^3^A_1_) isomer that could be located also has *C*_2v_ symmetry, which is clearly 9–18 kcal mol^−1^ higher in energy than the singlet ^1^A_1_ species.

**Fig. 2:**
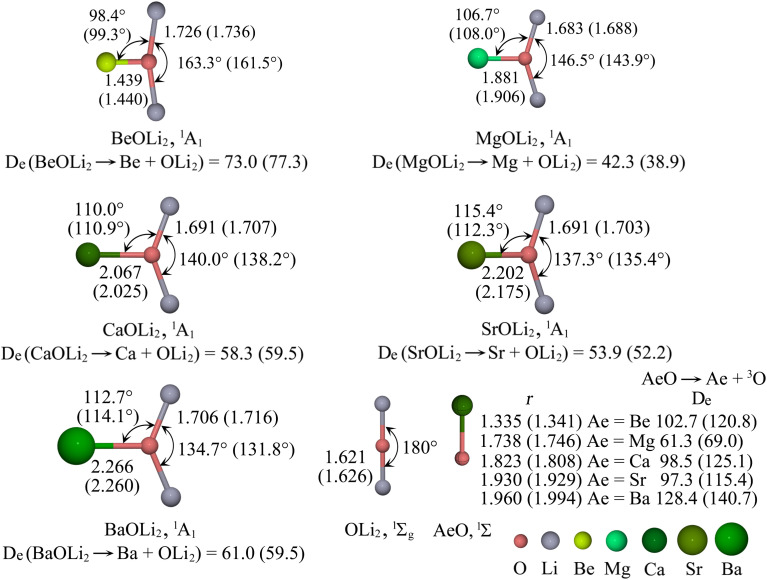
Calculated geometrical parameters and bond dissociation energies (*D*_e_, kcal mol^−1^) of the lowest-energy structure of AeOLi_2_ (Ae = Be, Mg, Ca, Sr, Ba) OLi_2_ and AeO computed at the CCSD(T)/def2-QZVPP level. The BP86-D3(BJ)/def2-QZVPP values are given in parentheses. The bond lengths are given in Å and the bond angles in degrees.

Fig. 2 shows the calculated bond lengths and angles of the singlet (^1^A_1_) structures of AeOLi_2_ and the computed bond dissociation energies (BDE) for breaking the Ae–OLi_2_ bond. The Ae–O bond lengths of the AeOLi_2_ adducts are clearly longer and the BDEs are smaller than those in diatomic AeO. The free OLi_2_ ligand is linear but it becomes bent in the AeOLi_2_ adducts with the bending angle becoming more acute from the lightest compound BeOLi_2_ to the heaviest adduct BaOLi_2_. Note that the BDEs for the Ae–OLi_2_ bond exhibit a zig-zag trend at both levels of theory from BeOLi_2_, which has the largest BDE, to BaOLi_2_. Both methods suggest that MgOLi_2_ has the lowest BDE of the Ae–O bonds.

Table 1 gives the calculated vibrational frequencies and IR intensities of the Ae–O stretching mode of the AeOLi_2_ adduct and free AeO. There is, as expected, a clear shift toward lower wave numbers for the adducts, which may be used as a helpful guide for the experimental studies. The frequency shift in the two methods is very similar. The complete vibrational spectrum of AeOLi_2_ is given in Table S1 of the ESI.‡

**Table:** Calculated vibrational frequencies *ν* (cm^−1^) of the Ae–O stretching mode in AeOLi_2_ and AeO (Ae = Be, Mg, Ca, Sr, Ba) and frequency shifts Δ*ν* at two levels of theory using the def2-QZVPP basis set. Vibrational intensities (km mol^−1^) are given in parentheses

Bond	BP86-D3(BJ)			CCSD (T)		
	AeOLi_2_	AeO	Shift Δ*ν*	AeOLi_2_	AeO	Shift Δ*ν*
Be–O	1102.8 (4)	1468.3 (3)	360.5	1114.8	1467.4	352.6
Mg–O	518.2 (0)	808.6 (16)	290.4	557.3	823.8	266.5
Ca–O	506.0 (19)	770.9 (89)	264.9	487.7	699.3	211.6
Sr–O	421.0 (17)	661.2 (83)	240.2	413.0	626.3	213.3
Ba–O	405.6 (32)	652.0 (143)	246.4	404.0	670.4	266.4

The above results indicate that the BP86 method gives very similar results to the CCSD(T) approach. Thus, we can confidently use BP86 for the bonding analysis of Ae–OLi_2_ bonds, which is the main topic of this work as discussed below. As a starting point, the atomic partial charges are presented and discussed, which were calculated using four different methods, namely the NBO, QTAIM, Hirshfeld, and Voronoi approaches. The results are shown in Table 2. The data given by the NBO and QTAIM methods differ significantly from those given by the other two approaches, particularly for the oxygen atom. The NBO and QTAIM methods suggest that the oxygen atom has a partial charge of nearly −2*e*, which in the case of QTAIM is even slightly larger than the value for a full valence shell. It is important to note that the NBO method treats only the (*n*)s-AOs, but not the (*n*)p-AOs of the alkali and alkaline earth atoms as true valence orbitals, while the (*n*)p-AOs are considered as Rydberg AOs, whose contribution is given less weight in the NBO algorithm. Since the covalent bond involves the s/p hybridization of the atoms, this means that the electronic charge of the covalent bonds is excessively assigned to the oxygen atom. The QTAIM method, on the other hand, uses the curvature of the charge distribution as a criterion for the assignment of atomic charges, which is known to lead to highly charged atoms. For example, the QTAIM charges for CO_2_ at the CCSD(T)/def2-QZVPP level are C (+2.73*e*) and O (−1.37*e*) which would mean that CO_2_ is mainly bonded by electrostatic attraction.^59^

Partial charges (*q*) and bond orders (*P*) of AeOLi_2_ (Ae = Be, Mg, Ca, Sr, Ba) computed at the BP86-D3(BJ)/def2-QZVPP level, using the CCSD (T) geometries

**Table:** 

Molecules	*q*_Ae_				*P*_Ae−O_	
	NBO	QTAIM	Hirshfeld	Voronoi^a^	Wiberg	Mayer
BeOLi_2_	0.24	0.36	−0.16	−0.18	0.23	1.05
MgOLi_2_	0.24	0.22	−0.06	−0.03	0.07	0.49
CaOLi_2_	0.33	0.31	0.04	0.07	0.30	0.84
SrOLi_2_	0.38	0.33	0.08	0.11	0.24	0.76
BaOLi_2_	0.52	0.46	0.12	0.17	0.30	0.86

a BP86-D3(BJ)/TZ2P.

**Table:** 

Molecules	*q*_O_			
	NBO	QTAIM	Hirshfeld	Voronoi^a^
BeOLi_2_	−1.88	−2.16	−0.56	−0.54
MgOLi_2_	−1.92	−2.02	−0.66	−0.69
CaOLi_2_	−1.79	−2.05	−0.64	−0.69
SrOLi_2_	−1.82	−2.05	−0.66	−0.71
BaOLi_2_	−1.80	−2.08	−0.64	−0.69

**Fig. 3:**
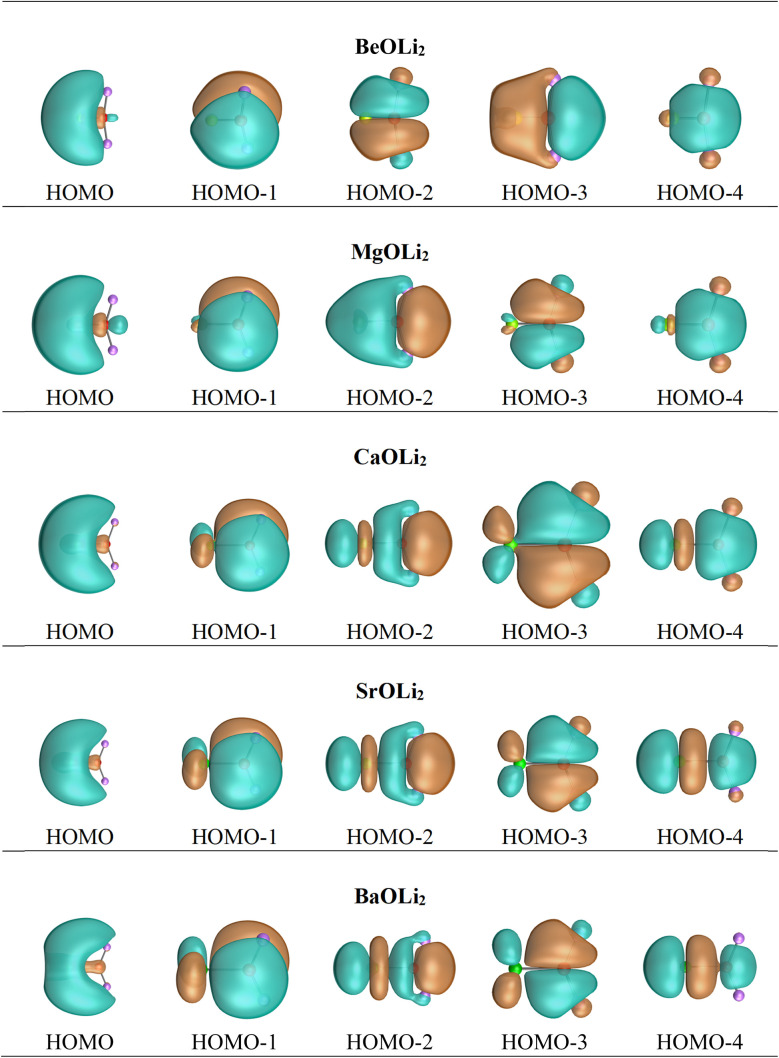
Shape of the five highest lying occupied Kohn–Sham MOs of AeOLi_2_ at BP86-D3(BJ)/def2-QZVPP.

In contrast, the Hirshfeld and Voronoi charges give a much smaller negative charge of a similar magnitude for oxygen in the AeOLi_2_ molecules between −0.56*e* and −0.71*e*. Both methods also suggest that the lighter Ae atoms Be and Mg are weak acceptors for the OLi_2_ ligand and that the heavy Ae atoms Ca, Sr, and Ba have only small positive charges in the molecules which are markedly smaller than those given by the NBO and QTAIM methods. We think that the Hirshfeld and Voronoi methods present a more faithful indication of the orbital overlap and the partial charges in the molecules. Fig. 3 shows the shape of the five highest-lying occupied orbitals of AeOLi_2_. It becomes obvious that there is a significant orbital overlap of the oxygen atom with Ae atoms and also with the Li atoms, which agrees with polar covalent bonds. The shape of the orbitals HOMO−1–HOMO−4 also shows that the heavier Ae atoms Ca, Sr, and Ba use their (*n*−1)d AOs for the covalent bonds.

Table 2 gives also the calculated bond order for the Ae–OLi_2_ bonds using the Wiberg method^44^ (WBO) and the Mayer partitioning approach (MBO).^45^ The Wiberg orders are based on the CNDO method which neglects the orbital overlap, whereas the MBO approach explicitly considers the AO overlap. It has been shown that this leads to drastically different values for polar covalent bonds.^60–62^Table 2 shows that the WBO values are much smaller than the MBO data, which provide a more faithful account of the covalent bonds. However, the MBO values must not be identified with the number of bonded orbitals. Note that polar single bonds have bond orders that may be much smaller than 1 and the MBO values of AeOLi_2_ between 0.49 and 1.05 do not rule out that there is more than one strongly polar orbital. More sophisticated methods are required to identify the covalent bond multiplicity of a chemical bond.

The charge distribution in a molecule determines the electric dipole moment, which is a vector property that provides useful information about the spatial orientation of the electronic charge. Table 3 shows that the calculated values of the dipole moments of AeOLi_2_ at the CCSD(T)/def2-QZVPP level are very large between 8.18 *D* for MgOLi_2_ and 3.78 *D* for BaOLi_2_, where the negative end of the dipole moment lies at the Ae atoms with the polarity Ae→OLi_2_. The BP86-D3(BJ)/def2-QZVPP values are a bit smaller but the overall trend is the same, except for BeOLi_2_, which has a slightly higher dipole moment than MgOLi_2_. A similar situation was reported for the valence isoelectronic anions AeF^−^ which possess large dipole moments with similar magnitude and a polarity Ae→F^−^.^24^ This was explained with the formation of a σ lone-pair orbital at atom Ae, whose center is located away from the nucleus of the atom.

**Table:** Calculated dipole moments *μ* (Debye) of AeOLi_2_ (Ae = Be, Mg, Ca, Sr, Ba) at two levels of theory using the def2-QZVPP basis set

Molecules	*μ*	
	BP86-D3(BJ)	CCSD(T)
BeOLi_2_	6.37	7.89
MgOLi_2_	6.21	8.18
CaOLi_2_	4.06	6.60
SrOLi_2_	3.54	5.80
BaOLi_2_	2.07	3.78

**Fig. 4:**
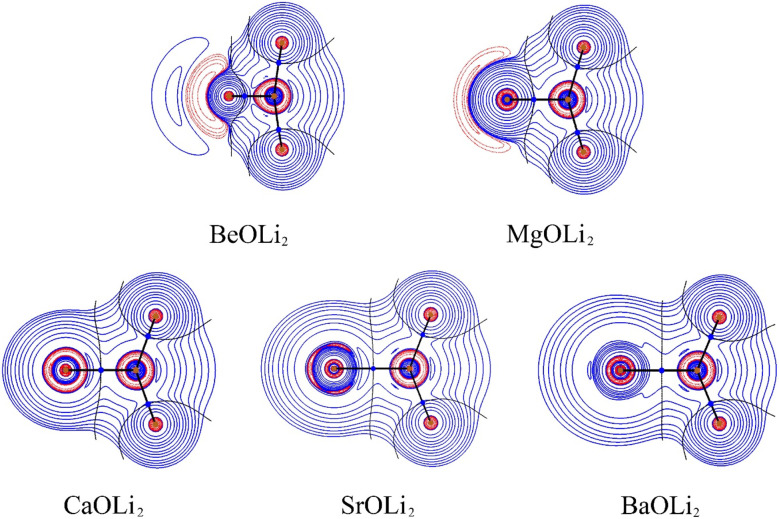
Laplacian distribution ∇^2^*ρ*(*r*) of AeOLi_2_ (Ae = Be, Mg, Ca, Sr, Ba) at the CCSD(T)/def2-QZVPP level. Red lines indicate the areas of charge concentration (∇^2^*ρ*(*r*) < 0), while blue lines show the areas of charge depletion (∇^2^*ρ*(*r*) > 0). The thick solid lines connecting the atomic nuclei are the bond paths. Blue dots are bond critical points (bcp). The thin lines which cross the bcp show the zero-flux surfaces in the molecular plane that separate the atomic basins. The alkaline earth metal atoms are given at the left.

Inspection of the spatial charge distribution in AeOLi_2_ reveals an apparently similar situation as in AeF^−^. Fig. 4 shows the Laplacian distribution of electron density, ∇^2^*ρ*(*r*) of AeOLi_2_ which exhibits a distinct area of charge concentration (red dashed lines) in the σ lone-pair region of BeOLi_2_ and MgOLi_2_. It is less visible in the Laplacian distribution of the heavier homologues, because the curvature of the electron-rich species is less pronounced. There are as expected Ae–O and O–F bond paths and bond critical points. Inspection of the occupied orbitals (Fig. 3) shows a very similar shape of the Ae σ lone-pair HOMO which occurs in all AeOLi_2_ species. A detailed analysis of the individual orbital components of the dipole moments in AeF^−^ showed that the electronic charge of the HOMO has a decisive influence on the overall dipole moment.^24^ The dipole moments of AeOLi_2_ show similar characteristics.

Very detailed information about the nature of the Ae–OLi_2_ bonds is available from EDA-NOCV calculations, which has proven to be a very powerful tool for bonding analysis in a variety of main-group compounds, transition-metal complexes as well as lanthanides and actinides.^55–58,63–66^ The focus of the EDA-NOCV method is the process of bond formation between the chosen fragments, which distinguishes it from most other methods like QTAIM,^34^ Interacting Quantum Atoms (IQA)^67^ and ELF^36^ that analyze the interaction between the atoms in the final molecule after the bond is formed. The choice of the electronic state and the charge of the fragments are crucially important for the results. It has been shown that the size of the orbital term Δ*E*_orb_ is a very helpful criterion to identify the most suitable fragments for the bonding analysis. The fragments that give the smallest Δ*E*_orb_ value are the best, as they change the least during bond formation, which proceeds along the path of bond dissociation/bond formation.^68–73^ We want to point out that the choice of the best fragments does not automatically identify the oxidation state of the atoms, because the oxidation state is related to the hypothetical charge of an atom if all of its bonds to other atoms were fully ionic. This may or may not be the same as the fragments which give the smallest Δ*E*_orb_ value.

In the case of AeOLi_2_, we employed several options for neutral charged fragments. It turned out that the neutral species Ae atom and OLi_2_ in their electronic singlet ground state clearly give the smallest absolute values for Δ*E*_orb_ and thus, they are used to analyze the bond formation of the Ae–OLi_2_ bond. The numerical results of EDA-NOCV are shown in Table 4. The EDA results using other fragments are given in Tables S2–S6 in the ESI.†

**Table:** EDA results of AeOLi_2_ at the BP86-D3(BJ)/TZ2P-ZORA level using Ae (ns^2^, ^1^S) + OLi_2_ (^1^A_1_) as interacting fragments. Energy values are given in kcal mol^−1^

Energy	Orbital interaction	Ae (ns^2^, ^1^S) + OLi_2_ (^1^A_1_)				
		BeOLi_2_	MgOLi_2_	CaOLi_2_	SrOLi_2_	BaOLi_2_
Δ*E*_int_		−81.0	−40.2	−60.9	−55.2	−65.0
Δ*E*_Pauli_		253.1	130.8	137.8	131.5	155.9
Δ*E*_disp_^a^		−1.7	−2.4	−2.7	−2.7	−2.8
Δ*E*_elstat_^a^		−189.0 (56.9%)	−114.2 (67.7%)	−126.9 (64.7%)	−121.4 (66.0%)	−138.7 (63.6%)
Δ*E*_orb_^a^		−143.4 (43.1%)	−54.4 (32.3%)	−69.2 (35.3%)	−62.6 (34.0%)	−79.4 (36.4%)
Δ*E*_orb(1)_^b^	Ae→OLi_2_ σ donation	−87.4 (60.9%)	−35.8 (65.8%)	−34.0 (49.1%)	−28.7 (45.8%)	−29.5 (37.2%)
Δ*E*_orb(2)_^b^	Ae←OLi_2_ σ backdonation	−19.3 (13.5%)	−7.0 (12.9%)	−14.9 (21.5%)	−15.3 (24.4%)	−24.3 (30.6%)
Δ*E*_orb(3)_^b^	Ae←OLi_2_ π backdonation	−18.6 (13.0%)	−5.9 (10.8%)	−10.3 (14.9%)	−9.2 (14.7%)	−12.3 (15.5%)
Δ*E*_orb(4)_^b^	Ae←OLi_2_ π backdonation	−16.2 (11.3%)	−4.9 (9.0%)	−8.6 (12.4%)	−7.8 (12.5%)	−10.3 (13.0%)
Δ*E*_orb(rest)_^b^		−1.8 (1.3%)	−0.7 (1.3%)	−1.2 (1.7%)	−1.5 (2.4%)	−2.8 (3.5%)

a The percentage contribution with respect to total attraction is given in parentheses.

b The percentage contribution in parentheses is given with respect to total orbital interaction.

Table 4 shows that the calculated total interaction energies Δ*E*_int_ of the Ae–OLi_2_ bonds are only slightly lower than and exhibit the same trend as the bond dissociation energies *D*_e_ (Fig. 2). This is because the two terms differ only in the geometrical deformation/relaxation of the OLi_2_ fragment, which requires very little energy. The attractive component of Δ*E*_int_ only has a small contribution from the dispersion interaction Δ*E*_disp_ and the major component comes from the electrostatic (Coulomb) attraction Δ*E*_elstat_, which provides 57–68% of the total attraction. This is reasonable, because the Ae–OLi_2_ bonds are very polar and the charge accumulation of the covalent interactions that comes from the interference of the wave functions is shifted toward the nucleus of the more electronegative atom. Note that the shift of the electronic charge from the midpoint of a bond toward one atom does not reduce but rather strengthens the quasiclassical Coulomb attraction, because the distance between the electronic charge in the bonding region and one nucleus becomes smaller. For a very insightful discussion, we refer to the literature.^74–76^ The electrostatic contribution to the interatomic interaction is sometimes termed as ionic bonding, which is a misleading expression. Ionic bonding occurs between charged fragments with negligible overlap, and is only found in ionic solids and ionic solution. There is no ionic bonding in molecules. The frequent occurrence of “ionic” bonding in molecules stems from the valence bond (VB) approach. The VB method has no explicit expression for a polar bond, which is mathematically described by a mixture of the terms for electron-pair bonding and ionic bonding.^77^ For details, we refer to a recent publication.^78^

**Fig. 5:**
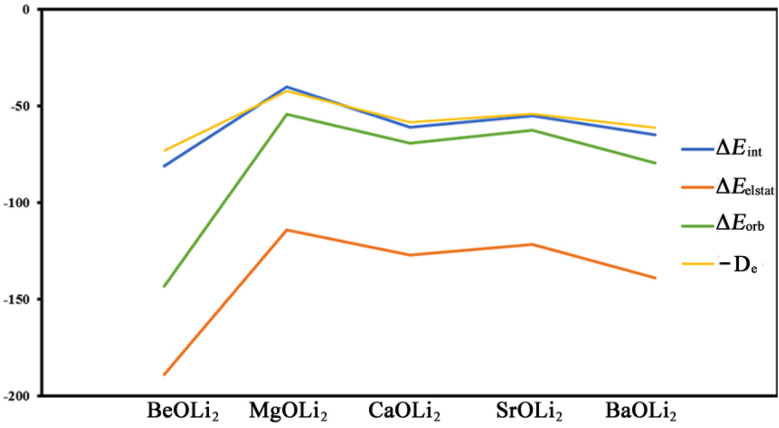
Trend of the bond dissociation energy *D*_e_ and the energy components of the EDA-NOCV calculations Δ*E*_int_ (total interaction energy of the frozen fragments), Δ*E*_elstat_ (electrostatic attraction) and Δ*E*_orb_ (orbital interaction).

It is useful to compare the trend of the EDA-NOCV energy components with the bond dissociation energies. Fig. 5 shows that the BDE values and the interaction energies Δ*E*_int_ between the frozen fragments exhibit the same zig-zag pattern. This means that the geometry relaxation of the OLi_2_ species has only a negligible effect on the relative bond strength of the Ae–OLi_2_ bonds. It is interesting to note that both attractive components Δ*E*_orb_ and Δ*E*_elstat_ also show the same zig-zag behavior. The bond strengthening/bond weakening may equally well be ascribed to the change in covalent bonding and electrostatic attraction. It is gratifying that the experimentally observed bond dissociation energies correlate well with the energy components of the EDA-NOCV calculations, which emphasizes the relevance of the method.

**Fig. 6:**
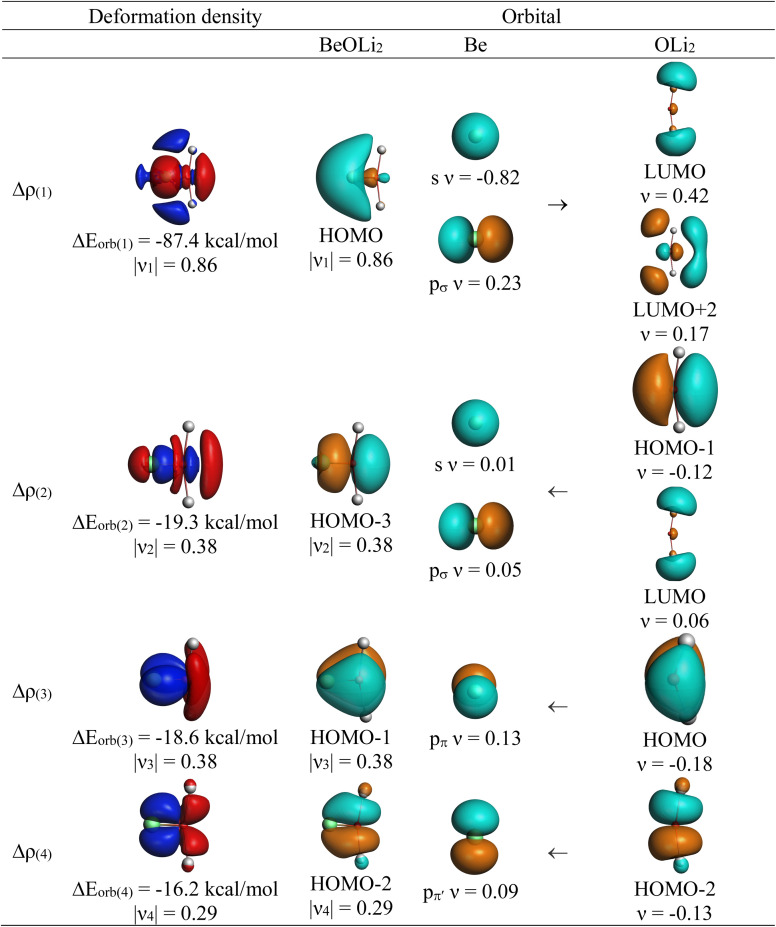
Plot of the deformation densities, Δ*ρ*_(1)–(4)_ shown as the sum of α and β electronic charges corresponding to Δ*E*_orb(1)–(4)_ and the related interacting orbitals in the singlet states of BeOLi_2_ at the BP86-D3(BJ)/TZ2P-ZORA level using Be (2s^2^, ^1^S) + OLi_2_ (^1^A_1_) as interacting fragments. The eigenvalues *ν* indicate the size of the charge flow. The direction of charge flow is red → blue. The isovalue for Δ*ρ*_(1)–(4)_ is 0.001 au.

**Fig. 7:**
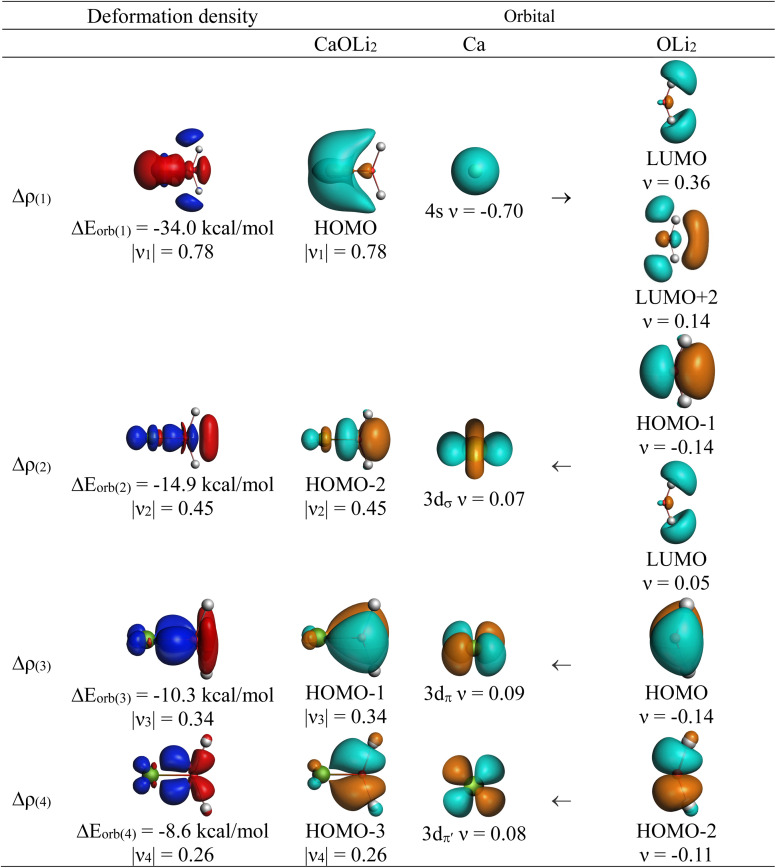
Plot of the deformation densities, Δ*ρ*_(1)–(4)_ shown as the sum of α and β electronic charges corresponding to Δ*E*_orb(1)–(4)_ and the related interacting orbitals in the singlet states of CaOLi_2_ at the BP86-D3(BJ)/TZ2P-ZORA level using Ca (4s^2^, ^1^S) + OLi_2_ (^1^A_1_) as interacting fragments. The eigenvalues *ν* indicate the size of the charge flow. The direction of charge flow is red → blue. The isovalue for Δ*ρ*_(1)–(4)_ is 0.001 au.

The most important information about Ae–OLi_2_ comes from the breakdown of the total orbital interaction Δ*E*_orb_ into pairwise contributions. Table 4 shows that there are four major terms Δ*E*_orb(1)_–Δ*E*_orb(4)_ which provide >96% of the covalent interactions. The other orbital term Δ*E*_orb(rest)_ comes from the relaxation of the fragment orbitals which are not directly involved in the Ae–OLi_2_ interactions. The nature of the pairwise orbital terms can be identified by examination of the associated deformation densities Δ*ρ* and the connected orbitals. They are shown for BeOLi_2_ and CaOLi_2_ in Fig. 6 and 7. The deformation densities and connected orbitals of MgOLi_2_, SrOLi_2_ and BaOLi_2_ are displayed in Fig. S1–S3 of the ESI.†

The strongest orbital term Δ*E*_orb(1)_ comes from Ae→OLi_2_ σ donation and the other three orbital interactions Δ*E*_orb(2)_ – Δ*E*_orb(4)_ are due to Ae←OLi_2_ backdonation with one σ component and two π components. This is a big difference to the results for the valence isoelectronic anions AeF^−^, where four components were also found, but all of them originate from the backdonation of Ae←F^−^.^23,24^ Unlike the F^−^ ligand, OLi_2_ has empty orbitals that can act as acceptor orbitals for donation from the occupied orbitals of the Ae atom. This leads to a different bonding situation, particularly in the lighter systems where Ae = Be, Mg. In AeF^−^, the two σ terms in BeF^−^ and MgF^−^ come from the concomitant σ donation and the polarization (hybridization) of the lone-pair AO of Ae, which are thus two components of the single σ backdonation Ae←F^−^. The bond multiplicity in BeF^−^ and MgF^−^ is, therefore, a triple bond Ae
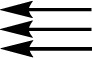
F^−^. In contrast, the neutral molecules BeOLi_2_ and MgOLi_2_ have genuine quadruple bonds where the occupied orbital of Ae atoms is a donor and the three vacant valence p AOs of Ae are acceptors featuring four dative bonds systems Ae
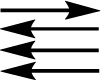
OLi_2_. The lighter systems with Ae = Be, Mg possess genuine quadruple bonds, because the valence orbitals of the metal atoms built strong dative interactions with the OLi_2_ fragment.

Quadruple bonds Ae
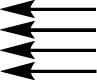
F^−^ were found for the anions with Ae = Ca, Sr, Ba because the heavier alkaline earth metals utilize their (*n*−1)d AOs for covalent bonding.^23,24^ Inspection of Fig. 7, S2 and S3† shows that the orbital interactions in the heavier molecules CaOLi_2_–BaOLi_2_ also involve the (*n*−1)d AOs of the metals as acceptor orbitals for the Ae←OLi_2_ σ and π backdonation Δ*E*_orb(2)_–Δ*E*_orb(4)_ but the strongest orbital term Δ*E*_orb(1)_ comes from Ae→OLi_2_ σ donation where the occupied (*n*)s AO of the atom Ae acts as a donor orbital. The heavier systems AeOLi_2_ where Ae = Ca, Sr, Ba have quadruple bonds Ae
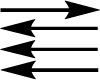
OLi_2_ like the lighter systems with Ae = Be, Mg.

**Fig. 8:**
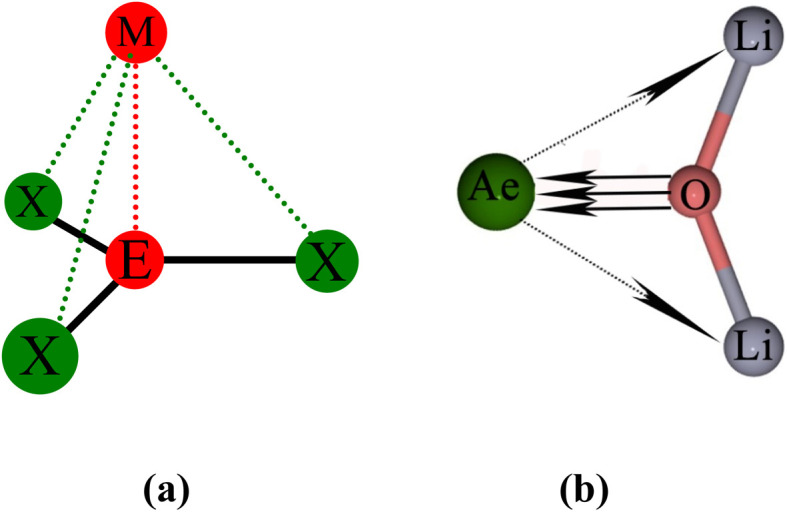
Schematic representation of (a) the proposed nature of the bonding in collective interactions where the red dashed line indicates Coulomb repulsion and the green dashed line indicates electrostatic attraction; (b) covalent bonding in AeOLi_2_ which consists of three (σ and 2π) dative interactions Ae
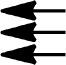
OLi_2_ and one bifurcated σ donation Ae→OLi_2_.

The strongest orbital interaction term Δ*E*_orb(1)_, which comes from Ae→OLi_2_ σ donation that does not occur in AeF^−^, shall be analyzed in more detail. Fig. 6, 7 and S1–S3† show that the acceptor orbitals LUMO and LUMO+2 of OLi_2_ are antibonding O–Li orbitals where the largest coefficients are at the lithium atoms. Inspection of the areas of charge depletion and charge accumulation Δ*ρ*_(1)_ shows that the area along the Ae–O axis undergoes charge depletion while the area of charge accumulation is actually found along the two Ae–Li bond axes. This holds for all molecules AeOLi_2_ (Ae = Be–Ba). This means that the strongest orbital interaction Δ*E*_orb(1)_ of AeOLi_2_ is rather an Ae–Li bond to both lithium atoms than an Ae–O bond. This is a very unusual situation that resembles the collective interactions recently proposed by Pendas and Foroutan-Nejad *et al.* as ‘exotic bonds’ in organometallic compounds.^79^ The relevance of collective interactions was disputed by Bickelhaupt, Sola and coworkers^80^ but it was supported by further work.^81–83^ A comparison of the nature of the collective interactions proposed by the authors with the bonding in AeOLi_2_ shows that there is only a structural similarity whereas the nature of the chemical bonds is very different from each other. This is illustrated in Fig. 8a, where the interatomic interactions between a metal atom M, an electron-poor atom E and electron-rich atoms X are shown. The figure is adapted from ref. 83. The authors suggest that there is electrostatic repulsion between atoms M and E (red dotted line) and the attraction comes from electrostatic attraction between M and atoms X. In contrast, the interaction between Ae and O atoms in AeOLi_2_ (Fig. 8b) comes from triple Ae
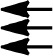
OLi_2_ dative bonding enhanced by electrostatic attraction. The feature of collective interaction comes from the unusual σ-backdonation Ae→OLi_2_ which is a covalent bond with two components that are directed toward the Li atoms. This type of bond is clearly different from the collective interactions proposed by Foroutan-Nejad and co-workers.^79,81–83^

It is noteworthy that the charge accumulation Δ*ρ*_(1)_ in the Ae–Li bonding region shown by the deformation densities does not lead to separate Ae–Li bond paths in the QTAIM analysis (Fig. 4). The Laplacian distribution ∇^2^*ρ*(*r*) gives the curvature of the density distribution and the absence of a bond path between two atoms does not prove that there is no covalent interaction. This has been shown previously.^84^ The strength of QTAIM analysis is that it provides detailed information about the overall electronic structure of a molecule after bond formation is complete, which can also be obtained from experiments. The weakness is that it gives no insight into the process of bond formation itself. There is also no direct information about the bond multiplicity. QTAIM also overlooks attractive interactions between atoms that are not strong enough to establish a critical bonding point.

The EDA-NOCV analysis reveals that the unusual σ bond with two components (collective interactions) occurs as the strongest component in the molecules AeOLi_2_, which leads to quadruple bonding in all compounds, even in BeOLi_2_ and MgOLi_2_. In contrast to Δ*E*_orb(1)_, the deformation densities Δ*ρ*_(2)_–Δ*ρ*_(4)_ of the other three orbital terms Δ*E*_orb(2)_–Δ*E*_orb(4)_ show that the charge accumulation (blue area) is along the Ae–O bond axis which indicates the formation of one σ and two π bonds. The Ae
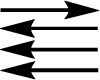
OLi_2_ quadruple bond has two σ and two π bonds where one σ bond is a collective bond between the Ae atom and Li_2_.

It is also interesting to analyze the two π backdonations Δ*E*_orb(3)_ and Δ*E*_orb(4)_ in more detail. Fig. 6 and 7 show that Δ*E*_orb(3)_ comes from the out-of-plane π_⊥_ backdonation Ae←OLi_2_, which is a bit stronger than the in-plane π_‖_ backdonation Δ*E*_orb(4)_. The former donation comes from the π lone-pair of the oxygen atom while the latter is an OLi_2_ bonding orbital with very small contributions of the Li atoms. But the donor orbitals of OLi_2_ in the orbital interactions of Δ*E*_orb(2)_–Δ*E*_orb(4)_ are not purely oxygen-based orbitals but also have some lithium valence AOs. The appearance of a vacant orbital of OLi_2_ in the Ae←OLi_2_ backdonation is due to the polarization of the occupied orbitals along the orbital interaction. The polarization of the fragment orbitals should be considered as part of the stabilizing orbital interactions, since it is caused by the bond formation.

The deformation densities associated with the orbital interactions Δ*E*_orb(1)_–Δ*E*_orb(4)_ explain nicely why there is still a σ lone-pair type area of charge accumulation at the Ae atoms although the valence electrons of the (*n*)s AO are engaged in the Ae→OLi_2_ donation that gives Δ*E*_orb(1)_. There is a concomitant charge donation in the opposite direction Ae←OLi_2_ due to the orbital interactions Δ*E*_orb(2)_–Δ*E*_orb(4)_.

The EDA-NOCV results demonstrate that there is a significant difference between the bond formation of F^−^ and valence isoelectronic OLi_2_ with Ae atoms in AeF^−^ and AeOLi_2_. The fluorine anion F^−^ may only be a donor whereas OLi_2_ may also be an electron acceptor, because it has vacant O–Li valence orbitals. The anion F^−^ donates electronic charge from three electron pairs (one σ and two π orbitals) to the three vacant valence orbitals of Be and Mg and it donates four electron pairs (two σ and two π orbitals) to four vacant valence orbitals of Ca, Sr, and Ba, which have an sd-valence space. OLi_2_ also donates three electron pairs (one σ and two π orbitals) to three vacant valence orbitals of all Ae atoms Be–Ba, but it forms a fourth dative bond through backdonation from the (*n*)s electron pair of the Ae atom to the vacant O–Li orbitals.

To ensure that the surprising quadruple binding in all systems AeOLi_2_ is not an artifact of the EDA-NOCV method, but is also suggested by other methods, a further analysis using adaptive natural density partitioning (AdNDP)^35^ was performed. The AdNDP method is a fundamentally different approach than the EDA-NOCV method and it is particularly well suited for assigning chemical bonding in the present case, where the contribution of the O–Li vacant orbitals to the chemical bonds is addressed. The AdNDP method was developed by Boldyrev and coworkers to give information about chemical bonding in delocalized systems.^35^

The search of 2c–2e orbitals involving the Ae–O moiety gave five orbitals with high occupation numbers (ONs) consisting of a lone pair at the oxygen atom, two Ae–O σ bonding orbitals and two π bonding orbitals. The numerical results are shown in Table 5 along with the shape of the orbitals for BeOLi_2_ and BaOLi_2_. The orbitals of the other species are similar and are shown in Fig. S4 of the ESI.† The four 2c–2e Ae–O bonding orbitals correlate nicely with the four interactions found in the EDA-NOCV analysis. The σ_2_ orbital and the two π orbitals with a very high ON close to 2 are related to the three orbital terms Δ*E*_orb(2)–(4)_ of the EDA-NOCV analysis whereas the σ_1_ orbital with a lower ON (1.32–1.73) is related to the Ae→OLi_2_ backdonation Δ*E*_orb(1)_. The latter orbital is more delocalized than the others, which becomes obvious due to the ON number for 4c–2e bonds (Table 5), but it is clearly identified as an Ae–OLi_2_ bonding orbital. The lower ON of the σ_1_ orbital is related to the orbital interaction Δ*E*_orb(1)_ where charge is donated to the LUMO and LUMO+2 of OLi_2_ (Fig. 6 and 7) which are mainly localized at Li.

AdNDP results of AeOLi_2_ at the BP86-D3(BJ)/def2-QZVPP level showing the occupation numbers (ONs) for 2c–2e (4c–2e) MO involving the Ae–O moiety. The shape of the two-center and four-center orbitals of BeOLi_2_ and BaOLi_2_. The orbitals of the other three systems are given in Fig. S4 of the ESI

**Table:** 

Molecules	σ_1_	σ_2_	π_1_	π_2_	Lone pair
BeOLi_2_	1.67 (2.00)	1.99 (2.00)	1.99 (2.00)	1.98 (2.00)	2.00 (2.00)
MgOLi_2_	1.73 (2.00)	1.99 (2.00)	1.98 (2.00)	1.97 (2.00)	2.00 (2.00)
CaOLi_2_	1.52 (2.00)	1.98 (2.00)	1.99 (2.00)	1.98 (2.00)	2.00 (2.00)
SrOLi_2_	1.50 (2.00)	1.98 (2.00)	1.99 (2.00)	1.98 (2.00)	2.00 (2.00)
BaOLi_2_	1.32 (2.00)	1.98 (2.00)	1.99 (2.00)	1.98 (2.00)	2.00 (2.00)

**Table:** 

σ_1_	σ_2_	π_1_	π_2_	Lone pair
**2c-2e Orbitals**				
		BeOLi_2_		
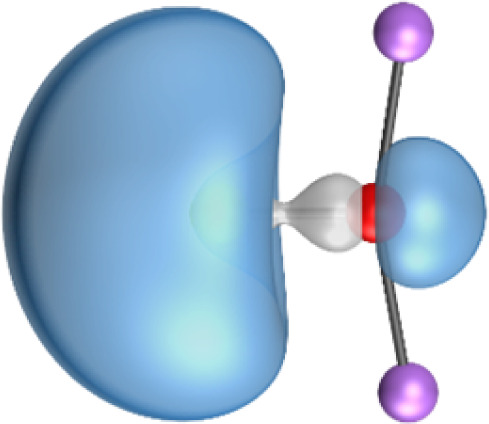	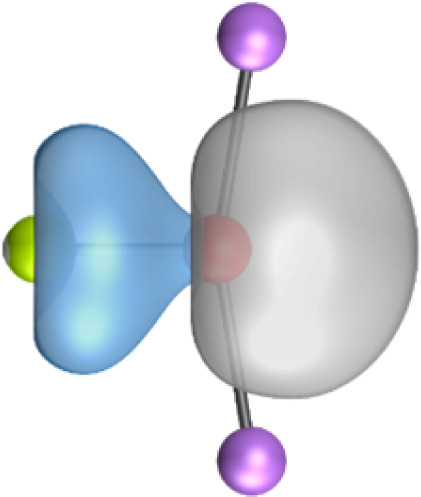	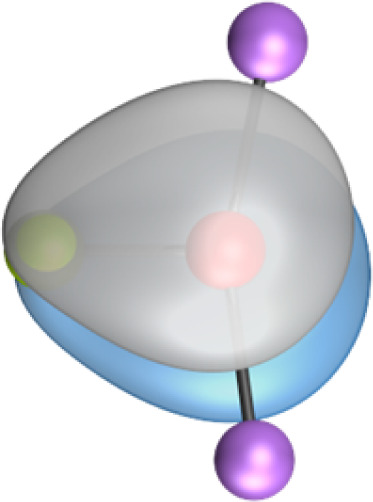	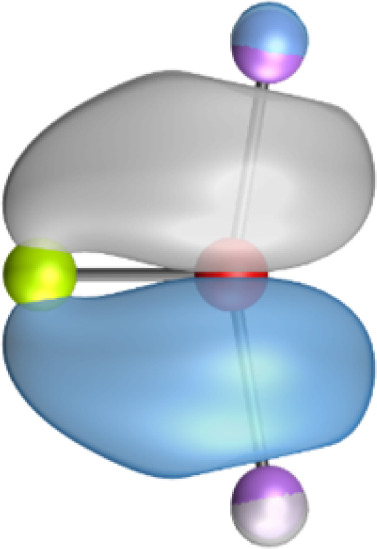	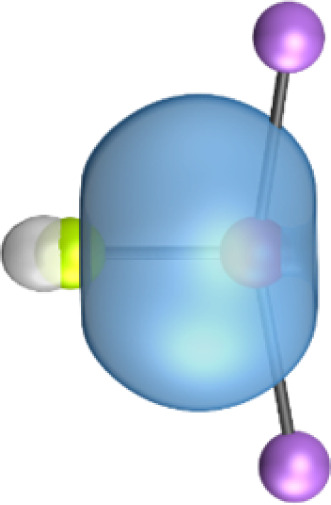
		BaOLi_2_		
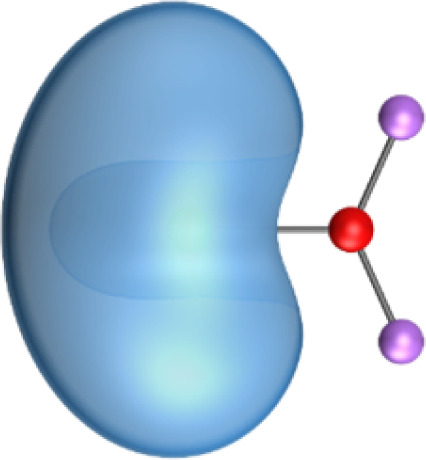	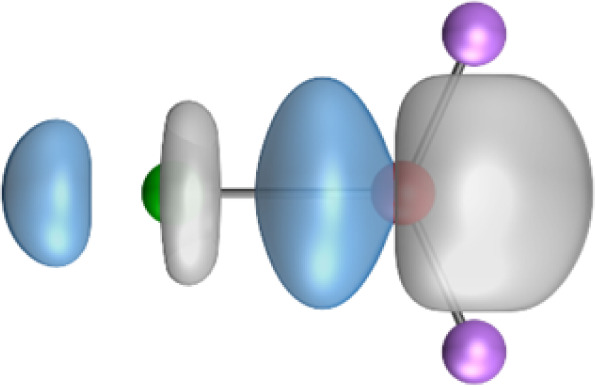	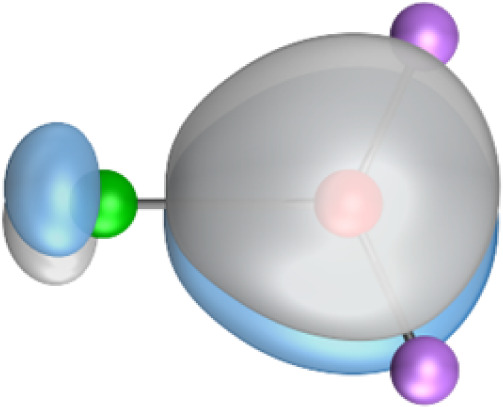	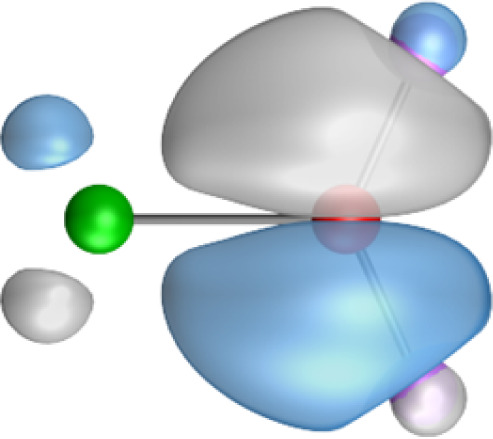	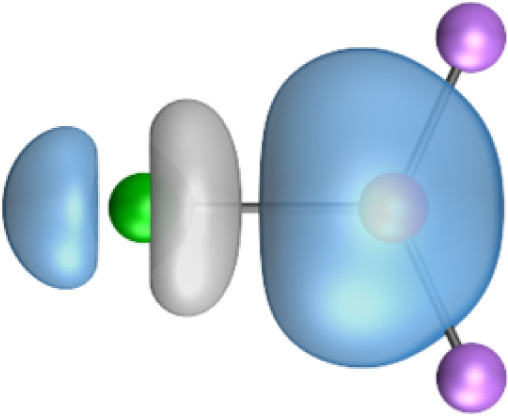
**4c-2e Orbitals**				
		BeOLi_2_		
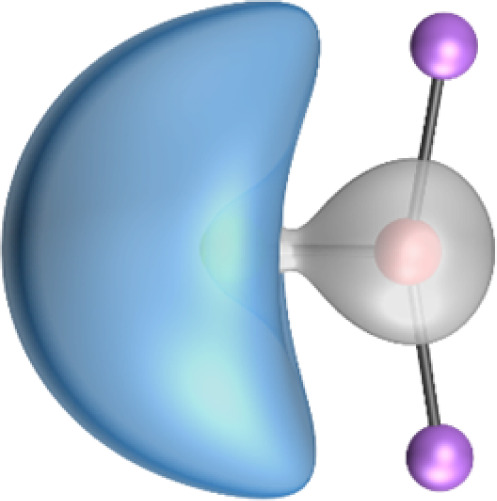	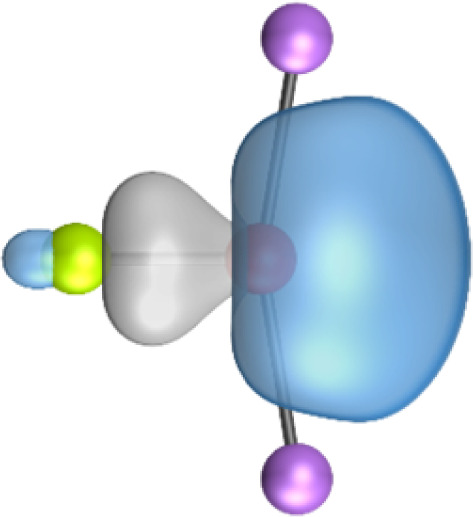	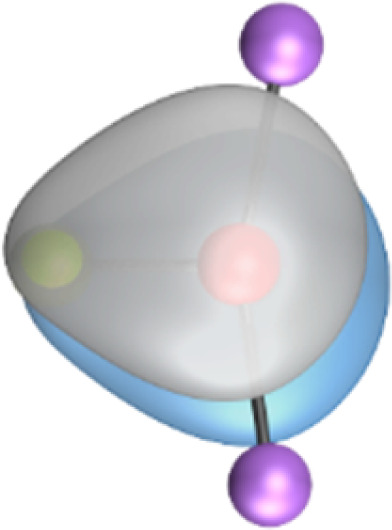	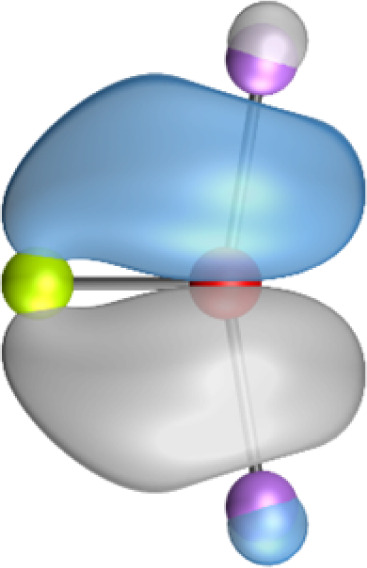	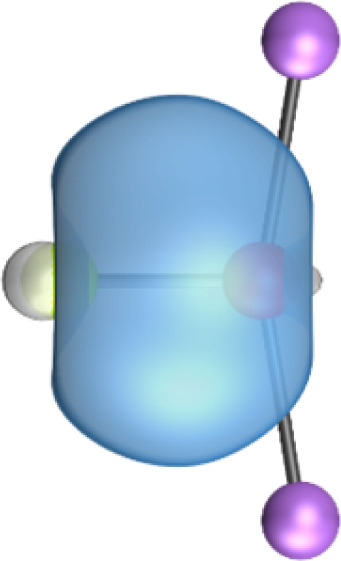
		BaOLi_2_		
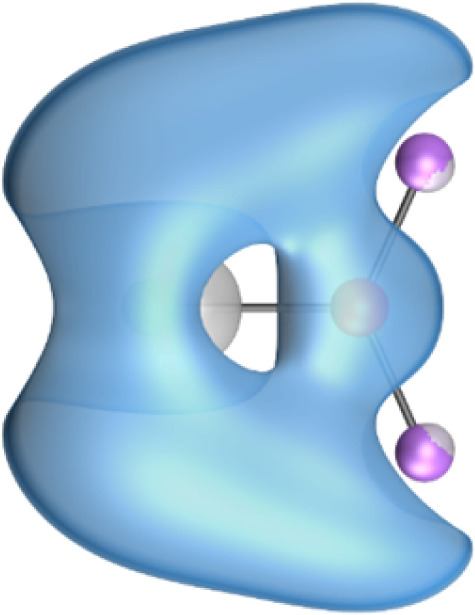	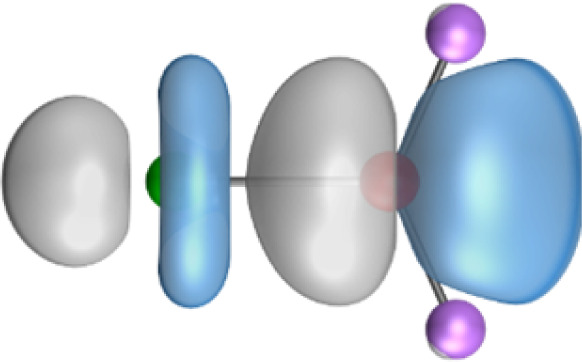	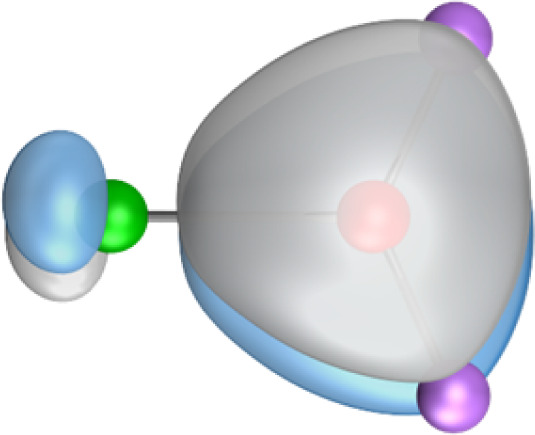	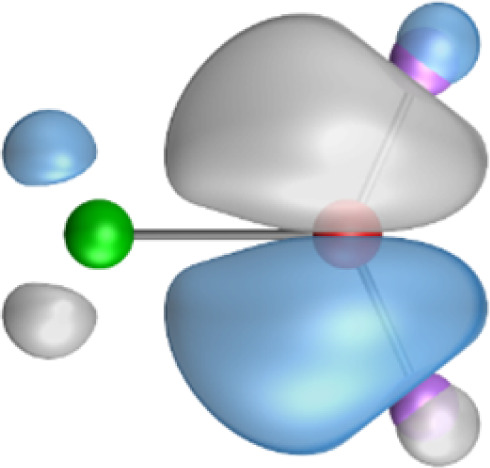	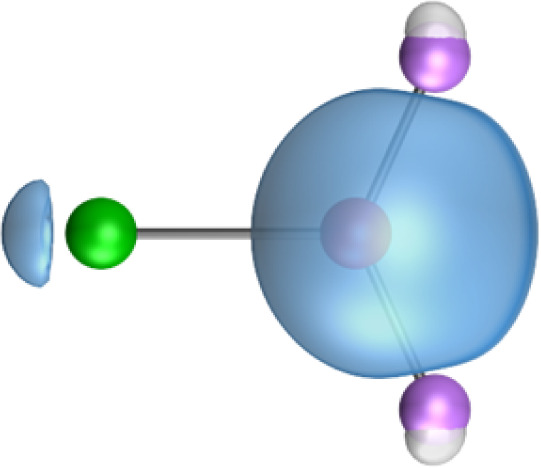

It is instructive to compare the 2c–2e AdNDP orbitals with the 4c–2e orbitals, which are also shown in Table 5. The shape of the orbitals σ_2_, π_1_, and π_2_ and the lone-pair are very similar since the 2c–2e orbitals have ON numbers of ∼2. The shape of the 4c–2e orbital σ_1_ clearly differs from that of the 2c–2e orbital σ_1_, particularly for BaOLi_2_, where the delocalization toward lithium becomes obvious. But the shape of the orbitals does not reveal that the covalent interaction takes place mainly between Ae and Li. The deformation densities *ρ*_1_ associated with the orbital interaction Δ*E*_orb(1)_, which are shown in Fig. 6, 7, S1–S3 give more direct evidence for the appearance of collective interactions due to the covalent bonding between the Ae atom and Li. But the AdNDP results support the conclusion of the EDA-NOCV analysis that all systems AeOLi_2_ have an Ae–O quadruple bond.

The suggestion of quadruple bonding Ae
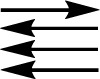
OLi_2_ is based on the energy contributions of the four orbital interactions. This is reasonable because chemical bonding in molecules is foremost an energy occurrence which comes from the interference of the wave functions. A related question concerns the associated charge distribution, which eventually emerges as a result of the chemical bond. Numerous methods have been developed to divide the total electronic charge of a molecule into atomic or electron pair regions, which provide important information about the electronic structure and the bonding situation. The QTAIM method and the Laplacian distribution mentioned above are examples where atomic basins are considered. A more fine-grained method is the ELF (Electron Localization Function) introduced by Becke and Edgecombe in 1990,^36^ which divides the total electronic charge into basins that can be associated with traditional chemical concepts such as bonding pairs, core electrons, and lone pairs. The concept of synapticity of the basin (or the attractor) was introduced by Savin *et al.*^85,86^ According to this approach, a disynaptic bonding basin, *V*(A,B), represents a covalent A–B bond, whereas monosynaptic basins, *V*(A), correspond to lone pairs of atom A in the Lewis representation of the valence electrons. The concept was further developed by Silvi for multicenter bonds who introduced the synaptic order.^87^ There are monosynaptic basins corresponding to electron lone pairs, disynaptic basins corresponding to conventional two-center bonds, trisynaptic basins corresponding to 3c–2e bonds, *etc.* In our previous work, we used the ELF method for analyzing the chemical bonds in AeF^−^, which has a maximum synaptic order of two.^24^ It is interesting to learn about the performance of the ELF method and the synaptic order in the AeOLi_2_ molecules, because the EDA-NOCV results suggest the occurrence of collective bonds between atoms that are not considered in a conventional Lewis model.

**Fig. 9:**
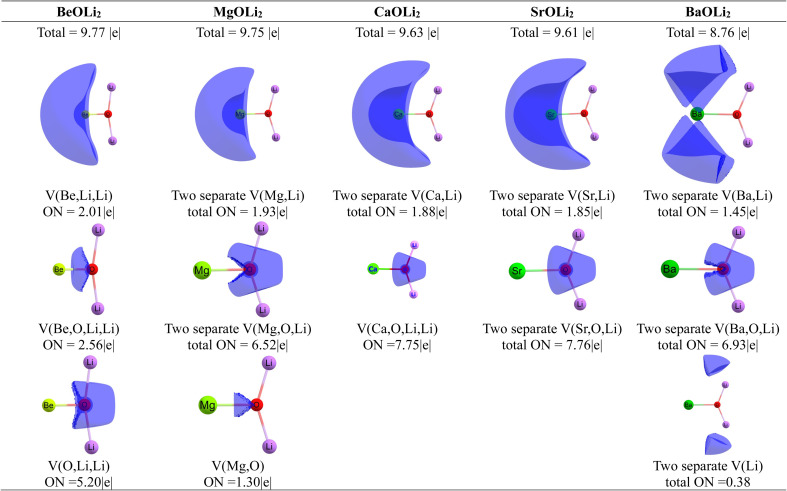
ELF calculation showing the synaptic basins and the occupation numbers (ONs) of AeOLi_2_ (Ae = Be–Ba) at CCSD(T)/def2-QZVPP. The contour line diagrams have an isovalue of 0.56*e* per a.u.

Fig. 9 shows the ELF results of the five molecules at the CCSD(T)/def2-QZVPP level. For BeOLi_2_, there are no monosynaptic or disynaptic basins. The calculation gives one trisynaptic basin *V*(Be,Li,Li) with a population of 2.01*e* and another trisynaptic basin *V*(O,Li,Li) with a population of 5.20*e*. In addition, there is a tetrasynaptic basin *V*(Be,O,Li,Li) that is populated by 2.56*e*. The ELF results for MgOLi_2_ are quite different. There are two separate diatomic basins *V*(Mg,Li) with a population of 1.93*e* and there is a diatomic basin *V*(Mg,O) which is populated by only 1.30*e*. There are furthermore two trisynaptic basins *V*(Mg,O,Li) with a total population of 6.52 *e*. The ELF results clearly show that the transformation of the synaptic basins into conventional Lewis structures without further analysis of the interatomic interactions is not possible for these molecules. The results also show that the lithium atoms are closely involved in the overall covalent bonding, which agrees with the analysis of the strongest Δ*E*_orb(1)_ term of the EDA-NOCV calculations featuring Ae→OLi_2_ σ donation between Ae and the Li atoms. We want to point out that the EDA-NOCV method uses the undisturbed electronic structures of Ae and OLi_2_ for the analysis of the Ae–OLi_2_ bonds, whereas the ELF approach considers the final electronic structure at the endpoint of bond formation. The two approaches are complimentary, but the EDA-NOCV results are more useful to identify the individual orbital interactions which provide the best Lewis structure for a molecule.

Fig. 9 shows also the ELF basins for the heavier AeOLi_2_ molecules where Ae = Ca, Sr. Ba. For CaOLi_2_, there are two separate disynaptic basins *V*(Ca,Li) with a total population of 1.88*e* and a tetrasynaptic basin *V*(Ca,O,Li,Li) that is populated by 7.75*e*. A similar situation is found for SrOLi_2_ with two separate diatomic basins *V*(Sr,Li) with a total population of 1.85*e* and two separate trisynaptic basins *V*(Sr,O,Li) that are populated by 7.76*e*. The finding of two separate trisynaptic basins in the latter molecule instead of a tetrasynaptic basin as in the calcium species is probably a numerical artefact. Somewhat different ELF results are calculated for BaOLi_2_. Fig. 9 shows that the two separate disynaptic basins *V*(Ba,Li) are populated by only 1.45*e* but they are complemented by two monosynaptic basins *V*(Li) with a total population of only 0.38*e*. The latter disynaptic and monosynaptic basins of the barium compound correspond to the diatomic basins of the calcium and strontium homologue. There are also two separate trisynaptic basins *V*(Ba,O,Li) but they are populated by only 6.93*e*, much less than the two separate trisynaptic basins *V*(Sr,O,Li) of the strontium molecule.

The ELF results of the heavier systems confirm the picture of the lighter homologues that there is a delocalized bonding interaction in AeOLi_2_ where the lithium atoms participate to a surprisingly large extent in the covalent bonding of the molecule. This can be explained by the EDA-NOCV results, where the strongest orbital interaction Δ*E*_orb(1)_ comes from Ae→OLi_2_ σ donation of the occupied (*n*)s^2^ AO into the vacant O–Li antibonding orbitals, which have the largest coefficient at Li. This leads to an unusual covalent Ae–Li_2_ interaction, which is not considered in the standard Lewis picture of chemical bonding. This is an important component of the quadruple bond between the Ae atoms and the OLi_2_ ligand which can be considered as an example of the recently introduced collective bonds. The description of the bonding situation with the formula Ae
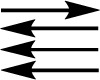
OLi_2_ comprises direct triple dative backbonding from oxygen to the Ae atom and σ bonding from Ae to Li_2_ which enhances the overall Ae–OLi_2_ attraction.

We also carried out ELF calculations of AeOLi_2_ using the electronic structures at the BP86-D3(BJ)/def2-QZVPP level. The results are very similar to values obtained at the CCSD(T)/def2-QZVPP level. They are shown in Fig. S5 of the ESI.† The atomic basins suggested by the two methods are the same with slightly different occupation numbers except that the BP86-D3(BJ)/def2-QZVPP calculation does not give monosynaptic basins *V*(Li) for BaOLi_2_ but a tetrasynaptic basin *V*(Ba,O,Li,Li) with an occupation of 7.55*e*.

## 4. Discussion

The results of the various methods for bond analysis clearly show that the description of the bonding situation in a molecule using standard Lewis formulae, which are a helpful model for describing the atomic structure and connectivity, is only a rough approximation for understanding the electronic structure of a compound. And it becomes clear that a true insight into the nature of interatomic interactions requires the use of multiple methods of charge and energy partitioning, the fundamentals and approximations of which must be known in order to provide meaningful information about the chemical bonds in a molecule. There are a ubiquitous number of publications in which a single method of bonding analysis is used – often without precise knowledge of the basic approximations of the method – and its results are then used as “evidence” for a seemingly authoritative interpretation of the chemical bonds in a molecule. This is particularly dangerous with molecules that have unusual chemical bonds and that differ from reference molecules.

The present molecules AeOLi_2_ (Ae = Be–Ba) and the nature of the Ae–OLi_2_ bonds are good examples for the above statement. The partial charges calculated using the Hirshfeld and Voronoi approaches indicate an approximate balance between the donation and back-donation between Ae and OLi_2_, with the back-donation Ae←OLi_2_ being slightly larger than the donation Ae→OLi_2_ for the lighter species with Ae = Be, Mg, while the reverse order is predicted for the heavier systems with Ae = Ca–Ba. The NBO and QTAIM methods suggest donation Ae→OLi_2_ for all systems, but both these methods have methodical deficiencies which make the results of the Hirshfeld and Voronoi approaches more reasonable.

The EDA-NOCV method suggests four distinct pairwise orbital interactions between Ae and OLi_2_ that clearly establish fourfold bonding between the two fragments. This is supported by the AdNDP approach, which transforms the electronic wavefunction into the most appropriate Lewis structure. Close examination of the four orbital interactions shows that the strongest component comes from Ae→OLi_2_ σ donation from the (*n*)s^2^ electron pair of Ae into vacant OLi_2_ orbitals, which have the largest coefficient at the Li atoms. Inspection of the associated charge deformation reveals that a covalent bond is present between the Ae atom and the lithium atoms, where the charge accumulation due to the interference of the wavefunction is along the two Ae–Li bond axes. This resembles the recently proposed collective interactions, where a covalent bond is formed between two atoms that are not directly connected when the molecule is sketched with a Lewis structure.

The remaining three orbital interactions come through Ae←OLi_2_ backdonation from energetically high-lying occupied MOs of OLi_2_ into vacant AOs of Ae. The latter AOs are the (*n*)p orbitals of Ae = Be, Mg while for the heavier Ae atoms Ca–Ba, the (*n*−1)d AOs are the acceptor orbitals. This shows that the heavier elements calcium, strontium and barium bind in molecules like transition metals. The occupied donor orbitals of OLi_2_ are mainly localized at oxygen with minor contributions at lithium. The four orbital interactions in AeOLi_2_ suggest some multicenter bonding, which is nicely reflected in the results of the ELF calculations. The ELF calculations of AeOLi_2_ give mainly trisynaptic and even tetrasynaptic basins for all systems. A proper sketch of the bonding situation in AeOLi_2_ which accounts for the quadruple bonding with four dative interactions is the formula Ae
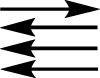
OLi_2_.

The results of this work might also stimulate experimental research on the catalytic properties of systems like AeOE_2_O (Ae = Be, Mg, Ca; E = Li, Na, K) where the bonding situation might be related to our systems.^88^ We plan to extend our studies in this direction.

## 5. Conclusion

The results of this work are summarized as follows.

• The lowest energy isomer of the AeOLi_2_ (Ae = Be–Ba) complexes calculated at the BP86-D3(BJ)/def2-QZVPP and CCSD(T)/def2-QZVPP levels has a *C*_2v_ geometry and a singlet (^1^A_1_) electronic ground state. The bond dissociation energy of the Ae–OLi_2_ bonds exhibit a zig-zag trend at both levels of theory from BeOLi_2_, which has the largest BDE (*D*_e_ = 73.0 kcal mol^−1^ at CCSD(T)), to BaOLi_2_. Both methods suggest that MgOLi_2_ has the lowest BDE (*D*_e_ = 42.3 kcal mol^−1^ at CCSD(T)) of the Ae–O bonds.

• The analysis of the chemical bonds with the EDA-NOCV method shows that the strongest component of the covalent interactions comes in all compounds from an unprecedented σ donor bond Ae→OLi_2_ where the (*n*)s^2^ lone-pair electrons of the Ae atom are donated to vacant O–Li_2_ antibonding orbitals having the largest coefficient at lithium. It is a covalent bond where the accumulation of the associated electronic charge is located at two positions above and below the Ae–OLi_2_ axis. This bifurcated component of orbital interactions is structurally related to the recently proposed collective bonding model, but exhibits a completely different type of bonding.

• There are also three dative bonds due to Ae
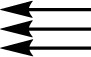
OLi_2_ backdonation which consists of one σ bond and two π bonds. The appearance of strong Ae→OLi_2_ σ donation leads to quadruple bonds Ae
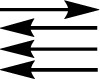
OLi_2_ for all AeOLi_2_ systems, even for the lightest species with Ae = Be, Mg. The valence orbitals of Ca, Sr, and Ba which are involved in the dative interactions are the (*n*)s and (*n*−1)d AOs whereas Be and Mg use their (*n*)s and (*n*)p AOs.

• The EDA-NOCV results are supported by AdNDP calculations which give four 2c–2e bonding orbitals. Three bonding orbitals have occupation numbers ∼2. One σ orbital has smaller occupation numbers between 1.32 and 1.73 due to the delocalization to the lithium atoms.

• The analysis of the electronic structure with the ELF method suggests multicenter bonds with mainly trisynaptic and tetrasynaptic basins, which also supports the results of the EDA-NOCV calculations.

• The calculation of the atomic partial charges by the Hirshfeld and Voronoi methods suggests that Be and Mg carry small negative charges in the lighter molecules whereas the heavier atoms Ca–Be have small positive charges. In contrast, the NBO and QTAIM methods give positive charges for all Ae atoms that are higher for Ca–Ba than those given by the Hirshfeld and Voronoi approaches.

• The molecules AeOLi_2_ have large dipole moments where the negative end is at the Ae atom with the polarity Ae→OLi_2_. The largest dipole moments are predicted for the lighter species BeOLi_2_ and MgOLi_2_ and the smallest value is calculated for BaOLi_2_.

• The calculation of the vibrational spectra shows a significant red-shift toward lower wave numbers for the Ae–OLi_2_ stretching mode with regard to diatomic AeO.

## Data availability

The data supporting this article have been included as part of the ESI.†

## Author contributions

S. P., Z. C., and G. F. conceived the project, wrote the draft, and finalized it, L. C. performed the calculations. L. C. and Y. L. analyzed the data. All authors took part in the discussions and approved the final version.

## Conflicts of interest

The authors declare no conflict of interest.

## Supplementary Material

SC-OLF-D4SC01979B-s001
