# Supplementary material for: Unusual quadruple bonds featuring collective interaction-type σ bonds between first octal-row atoms in the alkaline-earth compounds Ae[image]OLi2 (Ae = Be–Ba)
Source: Chem Sci. 2024 Jul 31;15(36):14705–20. doi: 10.1039/d4sc01979b (PMC11337084; doi:10.1039/d4sc01979b)
Supplement: SC-015-D4SC01979B-s001 [file SC-015-D4SC01979B-s001.pdf]

Supplementary Information (SI) for Chemical Science.
This journal is © The Royal Society of Chemistry 2024

Supporting Information For

Unusual Quadruple Bonds Featuring Collective Interaction-Type σ Bonds Between First Octal-Row Atoms in the Alkaline-Earth Compounds $Ae\substack{\rightarrow\\ \leftarrow\\ \leftarrow\\ \leftarrow}OLi_2$ (Ae = Be – Ba)

Li-juan Cui,[a#] Yu-qian Liu,[a#] Sudip Pan,*[a] Zhong-hua Cui*[a,b] and Gernot Frenking*[c,d]

[a]Institute of Atomic and Molecular Physics, Jilin University, Changchun 130023, China

E-mail: zcui@jlu.edu.cn (ZC); sudip@jlu.edu.cn (SP)

[b]Key Laboratory of Physics and Technology for Advanced Batteries (Ministry of Education), Jilin University, Changchun 130023, China

[c]Philipps-Universität Marburg, Hans-Meerwein-Strasse 4, D-35043 Marburg, Germany

[d]Institute of Advanced Synthesis, School of Chemistry and Molecular Engineering, Nanjing Tech University, Nanjing 211816, China.

E-mail: frenking@chemie.uni-marburg.de

[#]These authors contributed equally to this work.

Figures S1 – S5

Tables S1 – S8

Table S1. Calculated vibrational frequencies υ (cm^{-1}) of $AeOLi_2$ (Ae = Be, Mg, Ca, Sr, Ba) at CCSD(T) and BP86-D3(BJ) using the def2-QZVPP basis set. Vibrational intensities (km/mol) are given in parentheses.[a]

Mode	$BeOLi_2$	$MgOLi_2$	$CaOLi_2$	$SrOLi_2$	$BaOLi_2$
	BP86-D3(BJ)				
Li-O-Li out-of-plane bending	75.1 (5)	110.6 (26)	98.8 (1)	102.9 (1)	96.1 (2)
Li-O-Li in-plane bending	193.3 (37)	168.2 (38)	172.5 (6)	165.7 (5)	160.6 (2)
Li-O-Be bending	242.0 (326)	120.7 (153)	159.6 (106)	148.2 (89)	156.7 (58)
Li-O-Li symmetric stretching	606.9 (3)	685.0 (4)	665.4 (3)	668.2 (2)	650.1 (9)
Li-O-Li asymmetric stretching	749.9 (1)	840.2 (1)	784.3 (34)	790.4 (40)	755.2 (68)
Be-O stretching	1102.8 (4)	518.2 (0)	506.0 (19)	421.0 (17)	405.6 (32)
CCSD(T)					
Li-O-Li out-of-plane bending	84.9	124.8	116.8	115.4	102.3
Li-O-Li in-plane bending	204.6	186.7	185.2	174.8	174.3
Li-O-Be bending	241.0	106.4	131.8	135.8	156.5
Li-O-Li symmetric stretching	630.0	705.9	697.1	690.2	670.5
Li-O-Li asymmetric stretching	787.0	868.5	836.0	832.6	791.2
Be-O stretching	1114.8	557.3	487.7	413.0	404.0

[a] IR intensities at CCSD(T) are not available in the Gaussian program.

Table S2. The EDA results of the singlet state of $BeOLi_2$ cluster considering Be and OLi_2 in different charge and electronic states as interacting fragments at the BP86-D3(BJ)/TZ2P-ZORA level.

Energy	Be ($2s^2$, S) + OLi_2 (S)	Be ($2s^12p_x^1$, T) + OLi_2 (T)	Be^+ ($2s^1$, D) + OLi_2^- (D)	Be^{2+} ($2s^0$, S) + OLi_2^{2-} (S)
ΔE_{int}	-81.0	-189.0	-287.5	-778.5
ΔE_{Pauli}	253.1	148.0	173.2	80.6
ΔE_{disp}	-1.7	-1.7	-1.7	-1.7
ΔE_{elstat}	-189.0	-119.5	-266.8	-511.7
ΔE_{orb}	-143.4	-215.8	-192.2	-345.7

Table S3. The EDA results of the singlet state of $MgOLi_2$ cluster considering Mg and OLi_2 in different charge and electronic states as interacting fragments at the BP86-D3(BJ)/TZ2P-ZORA level.

Energy	Mg ($3s^2$, S) + OLi_2 (S)	Mg ($3s^1 3p_x^1$, T) + OLi_2 (T)	Mg^+ ($3s^1$, D) + OLi_2^- (D)	Mg^{2+} ($3s^0$, S) + OLi_2^{2-} (S)
ΔE_{int}	-40.2	-169.6	-214.5	-634.0
ΔE_{Pauli}	130.8	81.0	93.6	57.8
ΔE_{disp}	-2.4	-2.4	-2.4	-2.4
ΔE_{elstat}	-114.2	-76.3	-207.8	-463.2
ΔE_{orb}	-54.4	-171.8	-97.9	-226.2

Table S4. The EDA results of the singlet state of $CaOLi_2$ cluster considering Ca and OLi_2 in different charge and electronic states as interacting fragments at the BP86-D3(BJ)/TZ2P-ZORA level.

Energy	Ca ($4s^2$, S) + OLi_2 (S)	Ca^+ ($4s^1$, D) + OLi_2^- (D)	Ca ($4s^13d_{z^1}^{1}$, T) + OLi_2 (T)	Ca ($4s^13d_{yz}^1$, T) + OLi_2 (T)
ΔE_{int}	-60.9	-199.8	-164.2	-157.2
ΔE_{Pauli}	137.8	116.4	101.7	86.4
ΔE_{disp}	-2.7	-2.7	-2.7	-2.7
ΔE_{elstat}	-126.9	-214.4	-71.0	-76.0
ΔE_{orb}	-69.2	-99.2	-192.2	-164.9
Energy	Ca^{2+} ($4s^0$, D) + OLi_2^{2-} (D)			
ΔE_{int}	-544.7			
ΔE_{Pauli}	98.7			
ΔE_{disp}	-2.7			
ΔE_{elstat}	-455.4			
ΔE_{orb}	-185.3			

Table S5. The EDA results of the singlet state of $SrOLi_2$ cluster considering Sr and OLi_2 in different charge and electronic states as interacting fragments at the BP86-D3(BJ)/TZ2P-ZORA level.

Energy	Sr ($5s^2$, S) + OLi_2 (S)	Sr^+ ($5s^1$, D) + OLi_2^- (D)	Sr ($5s^1 4d_{yz}^1$, T) + OLi_2 (T)	Sr ($5s^1 4d_{z^2}^1$, T) + OLi_2 (T)
ΔE_{int}	-55.2	-185.0	-181.1	-165.6
ΔE_{Pauli}	131.5	116.3	93.3	100.4
ΔE_{disp}	-2.7	-2.7	-2.7	-2.7
ΔE_{elstat}	-121.4	-208.9	-78.4	-71.2
ΔE_{orb}	-62.6	-89.7	-193.2	-192.0
Energy	Sr^{2+} ($5s^0$, S) + OLi_2^{2-} (S)			
ΔE_{int}	-511.1			
ΔE_{Pauli}	106.9			
ΔE_{disp}	-2.7			
ΔE_{elstat}	-447.7			
ΔE_{orb}	-167.6			

Table S6. The EDA results of the singlet state of $BaOLi_2$ cluster considering Ba and OLi_2 in different charge and electronic states as interacting fragments at the BP86-D3(BJ)/TZ2P-ZORA level.

Energy	Ba ($6s^2$, S) + OLi_2 (S)	Ba^+ ($6s^1$, D) + OLi_2^- (D)	Ba ($6s^1 5d_{yz}^1$, T) + OLi_2 (T)	Ba ($6s^1 5d_{z^2}^1$, T) + OLi_2 (T)
ΔE_{int}	-65.0	-183.0	-152.8	-137.6
ΔE_{Pauli}	155.9	147.4	126.1	133.2
ΔE_{disp}	-2.8	-2.8	-2.8	-2.8
ΔE_{elstat}	-138.7	-225.6	-102.6	-88.4
ΔE_{orb}	-79.4	-102.1	-173.4	-179.6
Energy	Ba^{2+} ($6s^0$, S) + OLi_2^{2-} (S)			
ΔE_{int}	-485.3			
ΔE_{Pauli}	145.8			
ΔE_{disp}	-2.8			
ΔE_{elstat}	-460.2			
ΔE_{orb}	-168.1			

Table S7. Coordinates of singlet $AeOLi_2$ calculated at BP86-D3(BJ)/def2-QZVPP.

$BeOLi_2$			
Be	0.000000000000	0.000000000000	-1.212868000000
O	0.000000000000	0.000000000000	0.226749000000
Li	0.000000000000	1.713602000000	0.506246000000
Li	0.000000000000	-1.713602000000	0.506246000000
$MgOLi_2$			
Mg	0.000000000000	0.000000000000	1.146900000000
O	0.000000000000	0.000000000000	-0.758933000000
Li	0.000000000000	1.605234000000	-1.281891000000
Li	0.000000000000	-1.605234000000	-1.281891000000
$CaOLi_2$			
Ca	0.000000000000	0.000000000000	0.941164000000
O	0.000000000000	0.000000000000	-1.083554000000
Li	0.000000000000	1.594847000000	-1.692474000000
Li	0.000000000000	-1.594847000000	-1.692474000000
$SrOLi_2$			
Sr	0.000000000000	0.000000000000	0.660118000000
O	0.000000000000	0.000000000000	-1.514636000000
Li	0.000000000000	1.575581000000	-2.161235000000
Li	0.000000000000	-1.575581000000	-2.161235000000
$BaOLi_2$			
Ba	0.000000000000	0.000000000000	0.512031000000
O	0.000000000000	0.000000000000	-1.747656000000
Li	0.000000000000	1.566599000000	-2.448749000000
Li	0.000000000000	-1.566599000000	-2.448749000000

Table S8. Coordinates of singlet $AeOLi_2$ calculated at CCSD(T)/def2-QZVPP.

$BeOLi_2$			
Be	0.000000000000	0.000000000000	-1.202750000000
O	0.000000000000	0.000000000000	0.236092000000
Li	0.000000000000	1.707308000000	0.487043000000
Li	0.000000000000	-1.707308000000	0.487043000000
$MgOLi_2$			
Mg	0.000000000000	0.000000000000	1.124803000000
O	0.000000000000	0.000000000000	-0.756369000000
Li	0.000000000000	1.611745000000	-1.241114000000
Li	0.000000000000	-1.611745000000	-1.241114000000
$CaOLi_2$			
Ca	0.000000000000	0.000000000000	0.953293000000
O	0.000000000000	0.000000000000	-1.114014000000
Li	0.000000000000	1.589134000000	-1.692292000000
Li	0.000000000000	-1.589134000000	-1.692292000000
$SrOLi_2$			
Sr	0.000000000000	0.000000000000	0.663742000000
O	0.000000000000	0.000000000000	-1.537781000000
Li	0.000000000000	1.574796000000	-2.153324000000
Li	0.000000000000	-1.574796000000	-2.153324000000
$BaOLi_2$			
Ba	0.000000000000	0.000000000000	0.509631000000
O	0.000000000000	0.000000000000	-1.756815000000
Li	0.000000000000	1.574432000000	-2.414132000000
Li	0.000000000000	-1.574432000000	-2.414132000000

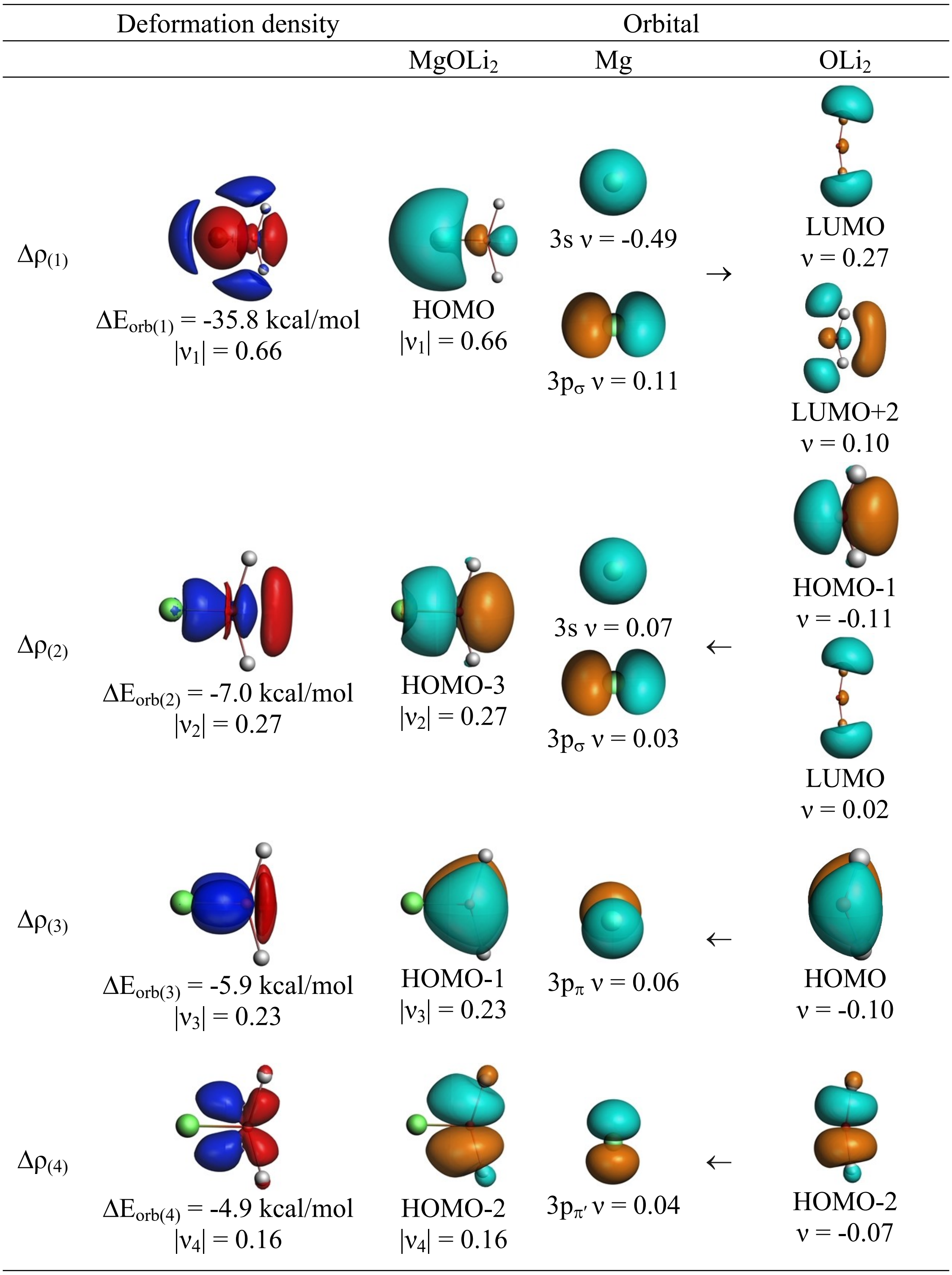

Figure S1. Plot of the deformation densities, $\Delta\rho_{(1)\text{-}(4)}$ shown as the sum of α and β electronic charge corresponding to $\Delta E_{orb(1)\text{-}(4)}$ and the related interacting orbitals in the singlet states of $MgOLi_2$ at the BP86-D3(BJ)/TZ2P-ZORA level using Mg ($3s^2$, S) + OLi_2 (S) as interacting fragments. The eigenvalues ν indicate the size of the charge flow, and the direction of charge flow is red → blue. The isovalue for $\Delta\rho_{(1)\text{-}(4)}$ is 0.001 au.

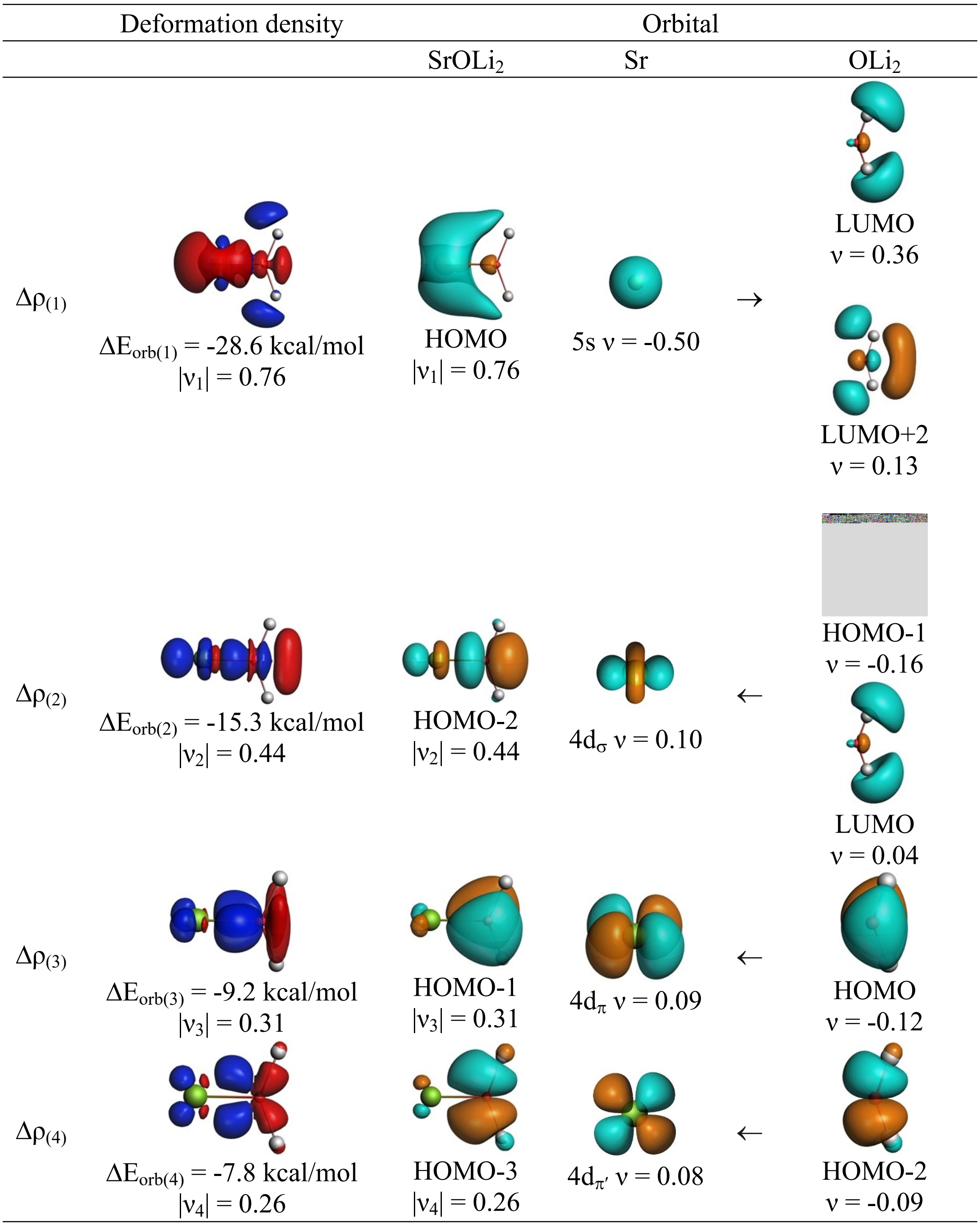

Figure S2. Plot of the deformation densities, $\Delta\rho_{(1)\text{-}(4)}$ shown as the sum of α and β electronic charge corresponding to $\Delta E_{orb(1)\text{-}(4)}$ and the related interacting orbitals in the singlet states of $SrOLi_2$ at the BP86-D3(BJ)/TZ2P-ZORA level using Sr ($5s^2$, S) + OLi_2 (S) as interacting fragments. The eigenvalues ν indicate the size of the charge flow, and the direction of charge flow is red → blue. The isovalue for $\Delta\rho_{(1)\text{-}(4)}$ is 0.001 au.

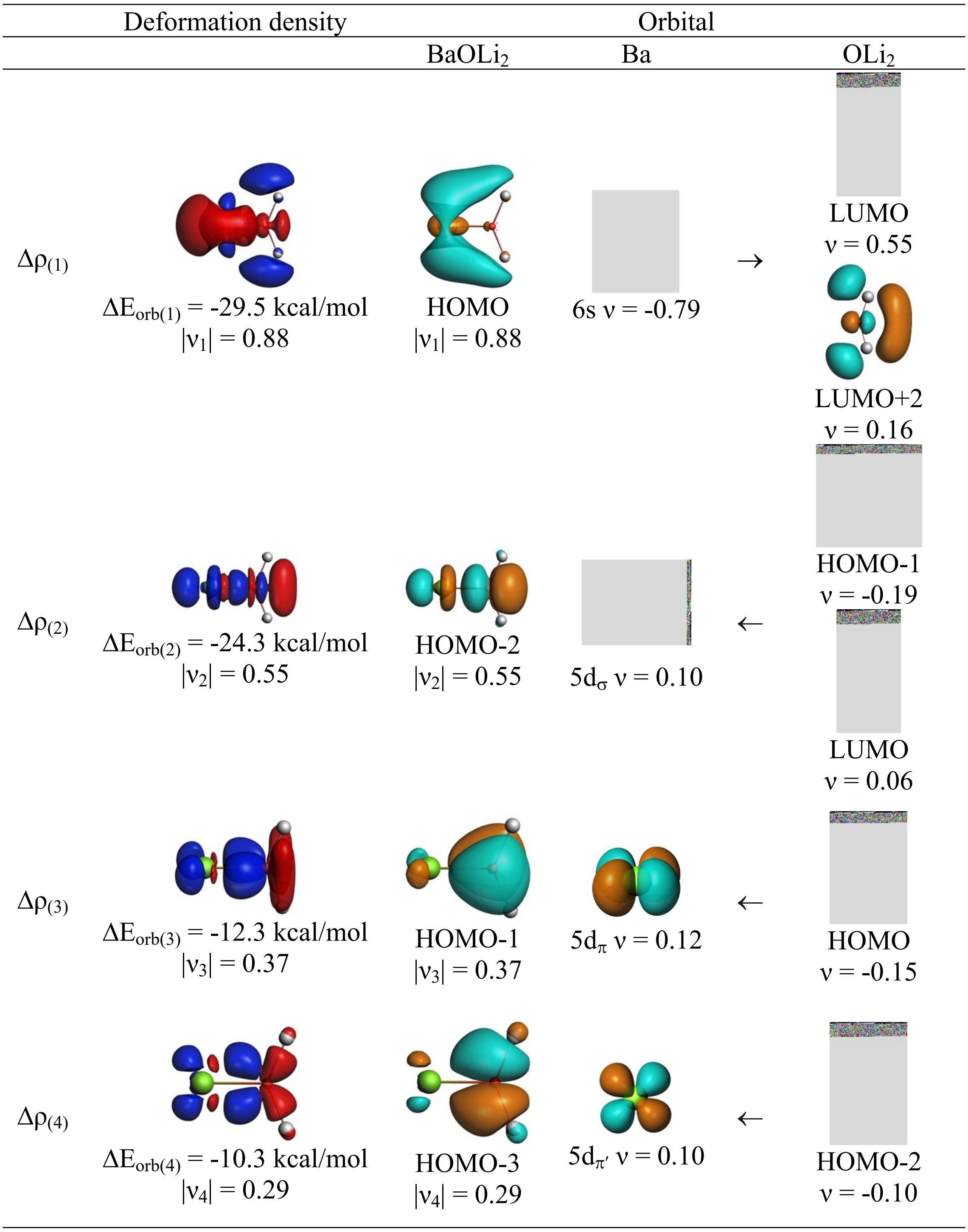

Figure S3. Plot of the deformation densities, $\Delta\rho_{(1)\text{-}(4)}$ shown as the sum of α and β electronic charge corresponding to $\Delta E_{orb(1)\text{-}(4)}$ and the related interacting orbitals in the singlet states of $BaOLi_2$ at the BP86-D3(BJ)/TZ2P-ZORA level using Ba ($6s^2$, S) + OLi_2 (S) as interacting fragments. The eigenvalues ν indicate the size of the charge flow, and the direction of charge flow is red→blue. The isovalue for $\Delta\rho_{(1)\text{-}(4)}$ is 0.001 au.

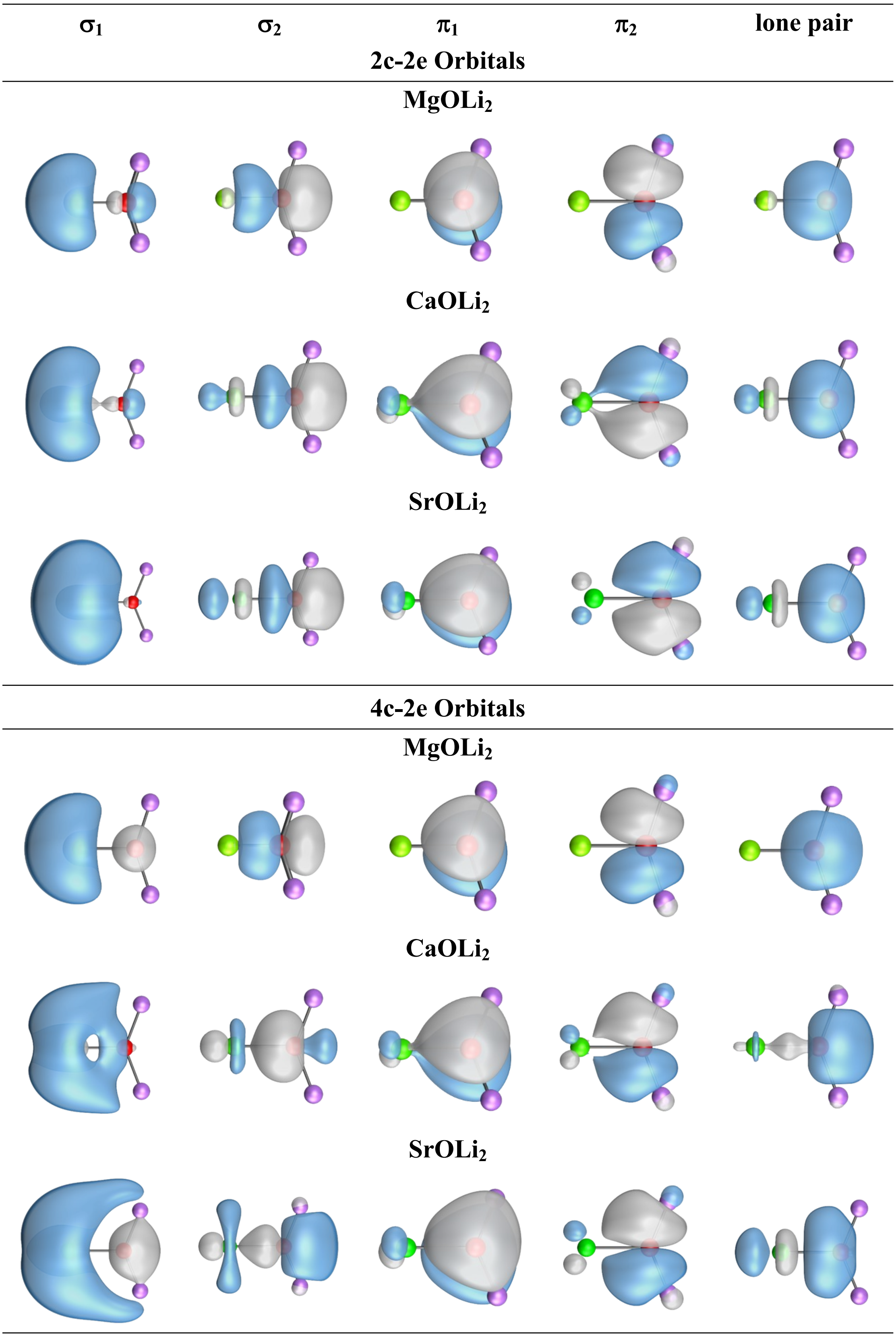

Figure S4. Shape of the AdNDP orbitals of $MgOLi_2$, $CaOLi_2$ and $SrOLi_2$.

$BeOLi_2$	$MgOLi_2$	$CaOLi_2$	$SrOLi_2$	$BaOLi_2$
Total = 9.75 |e|	Total = 9.73 |e|	Total = 9.50 |e|	Total = 9.50 |e|	Total = 9.39 |e|
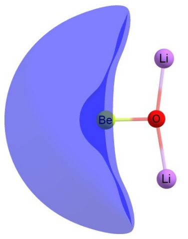	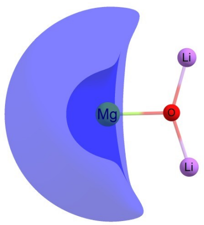	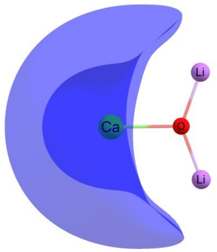	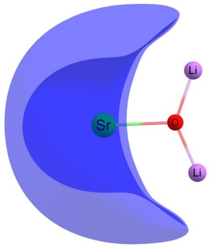	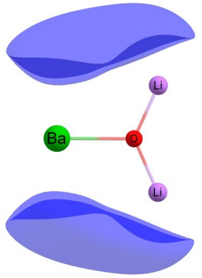
V(Be,Li,Li) ON =2.10|e|	Two separate V(Mg,Li) total ON = 1.96|e|	Two separate V(Ca,Li) total ON = 1.90|e|	Two separate V(Sr,Li) total ON = 1.87|e|	Two separate V(Ba,Li) total ON = 1.84|e|
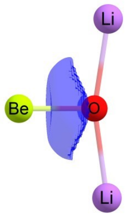	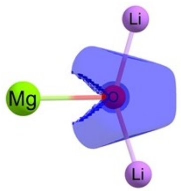	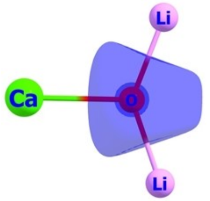	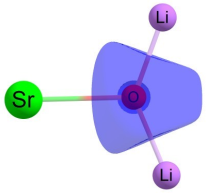	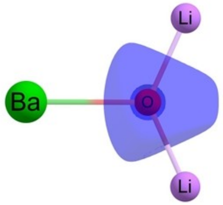
V(Be,O,Li,Li) ON =2.59|e|	Two separate V(Mg,O,Li) total ON = 6.76|e|	Two separate V(Ca,O,Li) total ON = 7.60|e|	Two separate V(Sr,O,Li) total ON = 7.63|e|	V(Ba,O,Li,Li) ON =7.55|e|
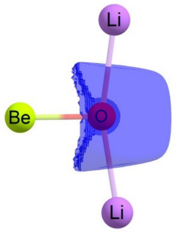	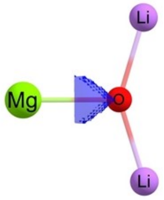			
V(O,Li,Li) ON =5.06|e|	V(Mg,O) ON =1.01|e|			

Figure S5. ELF calculation showing the synaptic basins and the occupation numbers ON of $AeOLi_2$ (Ae = Be – Ba) at BP86-D3(BJ)/def2-QZVPP. The contour line diagrams have an isovalue of 0.56e/a.u.
